# Membrane-Based Solar-Powered Brackish Water Treatment for Pastoral Livestock Watering in Arid Kazakhstan: A Techno-Economic Decision Framework

**DOI:** 10.3390/membranes16100331

**Published:** 2026-10-02

**Authors:** Timur Sh. Ustabaev, Gulzhan S. Alimbetova, Bakhtiyar Zh. Ismailov, Berik M. Zhaksybek, Ivan V. Basmanov, Anar Z. Batynova, Marzhan S. Kalmakhanova

**Affiliations:** 1 Kazakh Scientific Research Institute of Water Management LLP, Taraz 00008, Kazakhstan; timoha_85_85@mail.ru (T.S.U.); bakhtiyarismailov2016@mail.ru (B.Z.I.); beka.1999.w@gmail.com (B.M.Z.); ivan@mail.ru (I.V.B.); 2 Department of Chemistry and Chemical Technology, M. Kh. Dulaty Taraz University, Taraz 080012, Kazakhstan; g.a.kabdulova@gmail.com

**Keywords:** groundwater quality, groundwater treatment, membrane filtration, reverse osmosis, ultraviolet disinfection, ozonation, brackish groundwater, rural water supply, solar desalination, Kazakhstan

## Abstract

Access to safe water at pastoral watering points constrains livestock productivity across Kazakhstan’s extensive pasturelands, many of which remain underused because of inadequate water infrastructure. This review presents a decision framework for selecting off-grid groundwater treatment trains—membrane filtration, ultraviolet (UV) disinfection, and ozonation—according to source-water salinity, treatment capacity, operational constraints, and budget. Evidence from Kazakhstan, Central Asia, Australia, the Middle East, and sub-Saharan Africa indicates that photovoltaic (PV)-powered brackish water reverse osmosis (BWRO) combined with low-pressure UV disinfection is a strong baseline option when desalination and terminal microbial control are both required. Reported performance and cost ranges vary substantially with feed-water quality, scale, recovery, solar resource, storage strategy, and local logistics; they should therefore be treated as design inputs rather than universal values. Priority research needs include membrane fouling under continental climate extremes, bromate formation in local groundwaters, long-term solar-BWRO field performance, and the fate of antimicrobial resistance genes. The framework is intended to support site-specific technology selection rather than prescribe a single treatment train for all pastoral settings.

## 1. Introduction

Access to safe and sufficient water resources represents one of the fundamental prerequisites for sustainable agricultural development, particularly in regions characterized by arid and semi-arid climatic conditions. Arid land ecosystems occupy approximately 41% of the Earth’s land surface, are home to roughly 2.5 billion people who rely directly on dryland ecosystem services for their livelihoods, and support 50% of the world’s livestock [1]. The inherent hydrological vulnerability of these territories, compounded by progressive climate change and anthropogenic pressure, renders water scarcity not merely an environmental concern but a direct constraint on agricultural productivity and food security. Ensuring universal access to clean water and sanitation by 2030—as mandated by UN Sustainable Development Goal 6 (SDG 6)—remains one of the most critical and yet unmet targets of the global sustainability agenda, with urgency in rural and pastoral regions of the developing world [2].

At the global scale, freshwater availability per person is declining. The Food and Agriculture Organization reports that annual renewable freshwater resources per capita fell by more than 20% over the two decades preceding 2020 [3]. Agriculture accounts for nearly 70% of global freshwater withdrawals [4]. Within the agricultural sector, livestock production is highly dependent on reliable water supply, particularly where surface-water availability is limited or seasonal. Drinking-water quality can influence animal intake, growth, metabolic function, reproduction, and thermoregulation [5]. Consequently, both the quantity and chemical quality of water available at pastoral watering points are agronomic as well as water-security concerns.

These pressures are inseparable from the global food security imperative articulated in SDG 2 (Zero Hunger). Sustainable water management in pastoral systems is directly linked to food production capacity: deterioration of livestock water supply translates into reduced animal productivity, increased herd mortality during drought episodes, and accelerated degradation of rangeland vegetation cover [6]. At the same time, the deployment of energy-efficient, decentralized water treatment infrastructure in remote areas intersects with SDG 7 (Affordable and Clean Energy), as solar-powered purification systems represent a convergence point between water security and renewable energy access [2]. The restoration and sustainable management of arid and semi-arid land ecosystems, dependent in part on the availability of watering infrastructure, is further linked to SDG 15 (Life on Land) through its implications for combating desertification and land degradation [7].

The problem is especially acute in Central Asia, where the intersection of continental aridity, ageing water infrastructure, and climate variability has generated persistent water-security pressures. A major drought episode in 2021 affected large parts of the region and contributed to water shortages, crop losses, and livestock stress [8]. Kazakhstan, as the largest country in the region by territory, exemplifies these challenges at an exceptional scale. Of the country’s 186.4 million hectares of pastureland, more than 56.5% remains underused because of inadequate watering infrastructure and associated engineering constraints [9]. The same study reports that around 2 million head of livestock are exposed to water-supply risk in Southern Kazakhstan and that 69,836 rural residents lacked access to safe drinking water as of January 2024 [9].

The geographical extent of the water supply crisis in the pastoral zones of Central Asia is illustrated in Figure 1. The map delineates the distribution of arid (Aridity Index < 0.5) and semi-arid zones across Kazakhstan, Uzbekistan, Kyrgyzstan, Tajikistan, and Turkmenistan, overlaid with the spatial coverage of major pastoral regions, documented groundwater mineralization exceeding 1500 mg/L total dissolved solids, and locations facing a critical water supply deficit—defined as sites where pastureland utilization falls below 30% due to water shortage. The concentration of high-mineralization groundwater zones and critical supply deficit points within the core pastoral territories of southern and western Kazakhstan, the Aral Sea basin, and the Ferghana Valley underscores the regional scale of the treatment challenge addressed in this review.

The chemical composition of locally available groundwater constitutes an additional constraint. In the Almaty and Zhetysu regions, Tazhiyev et al. analysed 55 groundwater samples and reported that approximately 40% exceeded the mineralization benchmark applied in that study [9]. Because WHO does not establish a health-based guideline value for total dissolved solids (TDS), this result should be interpreted as evidence of a substantial mineralization/acceptability problem rather than as a universal WHO potability cut-off [10]. In arid and semi-arid pasturelands more broadly, dependence on wells and boreholes can concentrate livestock around a limited number of watering points, contributing to uneven grazing pressure and piosphere formation [11].

These realities underscore the pressing need for technically sound, economically viable, and ecologically safe water treatment solutions adapted to the operational constraints of remote pastoral territories. Over recent decades, substantial advances have been achieved in water purification chemistry and membrane engineering. Technologies including membrane filtration—encompassing microfiltration (MF), ultrafiltration (UF), nanofiltration (NF), and reverse osmosis (RO)—alongside ultraviolet (UV) photo-disinfection and ozonation (O_3_-based treatment), now provide the chemical and physical capacity to render brackish, mineralized, microbiologically contaminated, or otherwise non-potable water sources compliant with veterinary and potable water standards [12,13]. The progressive integration of these technologies with decentralized, photovoltaic-powered systems has further extended their applicability to locations without reliable grid electricity, making them directly relevant to the dispersed settlement patterns and remote watering points characteristic of arid pastoral zones [14,15].

Despite the growing scientific literature on individual treatment technologies, comprehensive reviews that systematically address their application to pastoral water supply infrastructure in arid zones remain limited. Most existing studies focus on either the physicochemical and engineering performance of treatment systems under controlled or urban conditions, or on hydrogeological resource assessment without addressing technology selection, integration, or pastoral-specific deployment constraints. The present review aims to bridge this gap by providing a critical analysis of the principal modern water treatment technologies applicable to pastoral watering infrastructure. The review examines the chemical mechanisms of contaminant removal, key operational parameters, comparative treatment efficiency, formation and management of disinfection by-products, energy and cost considerations, and the broader implications of technology adoption for pastureland productivity and sustainable rural development, with reference to the arid conditions of Kazakhstan and Central Asia.

## 2. Water Quality Challenges at Pastoral Water Sources in Arid Zones

### 2.1. Typology of Water Sources in Arid and Semi-Arid Pasturelands

In arid and semi-arid pastoral territories, the range of available water sources is substantially narrower than in humid agricultural regions, and each source category carries distinct physicochemical and microbiological characteristics that directly determine the feasibility and design of any treatment intervention. Three principal source types are encountered in practice: groundwater (shallow unconfined aquifers and deeper artesian systems), surface water (seasonal rivers, reservoirs, and ephemeral streams), and, to a lesser extent, treated or partially treated wastewater reused for agricultural purposes.

Groundwater constitutes the dominant and most operationally reliable source in many arid pastoral zones. Its chemical composition is governed by aquifer lithology, residence time, water–rock interaction, mixing, recharge, and evaporative concentration [16,17]. In Kazakhstan, groundwater is commonly extracted from boreholes and wells of variable depth and condition, and regional studies show pronounced spatial heterogeneity in mineralization and other water-quality parameters [9,11,18,19]. In the Almaty and Zhetysu survey of 55 samples, approximately 40% exceeded the mineralization benchmark used by the investigators [9]; this finding supports source-specific treatment assessment rather than a single regional TDS assumption.

Surface water sources, while seasonally abundant following snowmelt in mountain-fed systems, are subject to pronounced hydrological variability, elevated turbidity loads, and heightened microbiological contamination risk due to direct exposure to atmospheric deposition, runoff from grazed catchments, and the absence of natural filtration. In the lowland steppe and desert pastoral zones of Kazakhstan, surface water availability is frequently negligible for extended portions of the year, further reinforcing dependence on groundwater and increasing the strategic importance of treatment-based solutions [18].

The reuse of treated wastewater for pastoral water supply, while practiced in certain water-stressed regions of the Middle East and North Africa, remains at an early stage of regulatory and technical development in Central Asia. Its potential as a supplementary water source for livestock watering in peri-urban pastoral areas warrants systematic evaluation, provided that appropriate treatment standards are met and zoonotic contamination pathways are adequately controlled [20]. Although surface water and reclaimed wastewater are discussed for comparative purposes, groundwater remains the primary focus of this review because it represents the principal and most reliable water resource for livestock watering and rural water supply in arid regions.

### 2.2. Principal Physicochemical Contaminants

The physicochemical quality of groundwater in arid zones is shaped by a combination of geogenic and anthropogenic processes. The dominant geogenic mechanism is the dissolution of evaporite minerals—primarily halite (NaCl), gypsum (CaSO_4_·2H_2_O), and calcite (CaCO_3_)—which releases Na^+^, Cl^−^, Ca^2+^, SO_4_^2−^, and HCO_3_^−^ ions into solution. In structurally complex aquifer systems, ion exchange between calcium and sodium, cation enrichment through silicate weathering, and mixing of waters from horizons of differing mineralization further modulate the final ionic composition [16].

Elevated total dissolved solids (TDS) are a recurrent constraint in pastoral groundwater, but the degree of mineralization varies markedly among aquifers. Hydrogeological zoning across five regions of Southern Kazakhstan found that 92.5% of predicted exploitable groundwater resources had TDS ≤ 3 g/L, while also identifying pronounced regional deficits, particularly in Turkestan [17]. This ≤3 g/L criterion is a Kazakhstan resource-classification result and should not be presented as a WHO drinking-water threshold. Studies from Western Kazakhstan likewise document substantial spatial variation in groundwater hydrochemistry [21]. Treatment selection should therefore be based on measured source-water chemistry and intended use rather than on a universal regional TDS cut-off.

Beyond salinity, sulphate (SO_4_^2−^), nitrate (NO_3_^−^), fluoride (F^−^), iron (Fe^2+^/Fe^3+^), and manganese can constrain groundwater use. High sulphate may reduce palatability and, at sufficiently high concentrations, cause osmotic diarrhoea in susceptible livestock [22,23]. Nitrate contamination is associated with agricultural inputs and decomposing organic matter and can pose toxicological risk through conversion to nitrite in ruminants [22,24]. A recent study from the Almaty region demonstrated the relevance of nitrate exposure and source attribution in Kazakhstan groundwater [25]. Fluoride limits should be assessed against drinking-water standards where the same source serves people [10]. Iron and manganese are mainly operational concerns at typical groundwater concentrations because oxidation products can cause aesthetic problems, deposits, biofilm development, and membrane fouling [12,16].

Anthropogenic contamination pathways—although often secondary to geogenic mineralization in remote pastoral zones—remain important where wells are poorly protected, or agricultural inputs are intensive. In the Zhambyl Region, groundwater is widely used, and a substantial fraction of sampled sources did not meet drinking-water expectations for salinity, hardness, or microbiological quality [11]. Outdated or poorly protected well infrastructure can create pathways for surface-derived contamination of shallow aquifers [18]. These risks reinforce the need for source-specific hydrochemical and microbiological characterization before treatment selection.

### 2.3. Microbiological Hazards

Microbiological contamination of pastoral water sources constitutes a distinct and often underappreciated risk category, particularly in settings where livestock are watered directly from open wells, ponds, or poorly protected boreholes. The principal indicator organisms of concern are total coliforms, thermotolerant (faecal) coliforms, and *Escherichia coli*, whose presence signals faecal contamination and the potential co-occurrence of enteric pathogens of veterinary and public health significance [23].

Among the zoonotic pathogens of greatest concern in pastoral water systems are *Cryptosporidium parvum* and Giardia duodenalis, both protozoan parasites capable of completing part of their life cycle in livestock hosts and of persisting in water as environmentally resistant oocysts and cysts, respectively. A range of potentially waterborne zoonotic pathogens—including *Salmonella* spp., *E. coli* O157:H7, *Campylobacter* spp., and *Cryptosporidium parvum*—can be transmitted through drinking water contaminated by livestock faecal matter, which contributes significant levels of faecal indicator organisms to water sources used for both animal and human consumption [26].

The microbiological risk is compounded by the physical configuration of pastoral watering infrastructure. Open earthen ponds and unlined water holes are particularly vulnerable to faecal loading from congregating livestock, and the formation of characteristic piospheres—zones of intensive grazing and defecation radiating outward from water points—creates persistent pathways for faecal contamination of both surface and shallow groundwater [25]. The absence of residual disinfection in untreated rural water supplies means that any recontamination occurring during storage or distribution cannot be controlled at the point of use, a critical limitation acknowledged in the WHO framework for safe water supply in low-resource settings [10].

### 2.4. Regulatory Standards for Livestock Watering and Rural Drinking Water

Water-quality assessment for pastoral applications should distinguish between requirements for livestock watering and those for human drinking water when a source serves both purposes. Livestock tolerance depends on species, age, diet, climate, and acclimation [21,22]. WHO does not establish a health-based guideline value for TDS; instead, TDS, chloride, sulphate, iron, pH, and turbidity are interpreted largely through acceptability and operational considerations, while health-based values apply to constituents such as nitrate and fluoride [10]. For livestock, salinity should be interpreted together with sulphate, nitrate, and other constituents rather than from TDS alone [21,22]. Table 1 summarises the principal guidance and national limits used in this review.

Kazakhstan’s drinking-water requirements are established through national sanitary and epidemiological regulations, including Order No. 26 of 20 February 2023 [27]. For livestock watering, FAO guidance and the Australian and New Zealand livestock drinking-water guidance provide useful international reference points [21,22]. Where one installation supplies both livestock and people, the human drinking-water requirements should govern the potable stream, while a separate livestock stream may be designed to an appropriate species-specific target where regulations permit.

For livestock watering, international guidance allows substantially higher salinity than is normally acceptable for human drinking water, but tolerable concentrations remain species- and context-specific [21,22]. The Australian and New Zealand Livestock Drinking Water Guidelines provide species-differentiated guidance for salinity, nitrate, sulphate, and other constituents [22]. Where one source serves both livestock and people, the human drinking-water requirements govern the potable-water stream, whereas livestock-only treatment targets should be established from the complete ion profile, animal species, climate, diet, and acclimation.

Table 2 subsequently summarises selected source-specific groundwater-quality findings from Kazakhstan without combining non-comparable datasets into synthetic regional concentration ranges.

## 3. Membrane Filtration Technologies

### 3.1. Classification and Operating Principles

Existing reviews of membrane desalination performance and economics are concentrated overwhelmingly at municipal or industrial scale (hundreds to thousands of m^3^/day, grid-connected), leaving a gap in quantified guidance for the micro-scale, off-grid, solar-powered configurations (10–25 m^3^/day) characteristic of remote pastoral watering points. This section addresses that gap directly, developing membrane technology selection criteria specific to this deployment scale. Pressure-driven membrane filtration encompasses a spectrum of processes differentiated by membrane pore size, operating pressure, and the physicochemical mechanisms governing solute rejection. In order of increasing selectivity and operating pressure, the principal technologies are: microfiltration (MF), ultrafiltration (UF), nanofiltration (NF), and reverse osmosis (RO). Each occupies a distinct position in the treatment train and addresses a specific category of contaminants, which determines both its standalone applicability and its function within integrated multi-barrier systems [12].

In the context of pastoral water supply, the critical operational distinction lies between technologies capable of reducing ionic mineralization and those that are not. MF and UF provide effective barriers against particulate matter, protozoa, bacteria, and viruses but do not reduce TDS or dissolved ions—a fundamental limitation in brackish groundwater sources characteristic of arid Kazakhstan, where TDS frequently ranges from 1500 to 5000 mg/L [11,17]. NF and RO address this limitation through charge-based and size-based exclusion of dissolved species, with RO achieving near-complete rejection of all ionic contaminants and constituting the technology of choice for source waters with TDS exceeding 3000 mg/L [29]. The key operational parameters for each technology—pore size, operating pressure, energy consumption, contaminant rejection profile, and primary pastoral application—are summarised in Table 3.

Where full desalination is not required—for example, where source water TDS falls in the range of 1000–3000 mg/L—blending of UF permeate with NF/RO permeate in appropriate proportions provides a cost-effective means of achieving target mineralization while reducing the volume of water processed by high-pressure membranes and thereby lowering overall energy demand. For cattle watering specifically, where TDS targets are less stringent than for human drinking water (FAO limit ≤ 3000 mg/L under normal conditions [22,23], this approach can substantially reduce CAPEX and OPEX relative to full-stream RO treatment.

### 3.2. Physicochemical Basis of Contaminant Rejection

Transport through RO membranes is commonly described by the solution-diffusion framework: water flux increases with the effective pressure driving force, whereas salt transport is governed primarily by solute partitioning, diffusion, and concentration gradients through the selective layer [29,30]. This framework explains why increasing transmembrane pressure can raise water flux without a proportional increase in salt passage. RO and NF are also used to remove many dissolved organic and emerging contaminants, although rejection is compound- and membrane-specific rather than universally complete [31].

For NF membranes, solute rejection is governed by the combined operation of steric (size) exclusion, Donnan electrostatic exclusion, and dielectric exclusion—described within the DSPM-DE model framework [32]. This combination yields rejection rates of 85–98% for divalent ions (Ca^2+^, Mg^2+^, SO_4_^2−^) while permitting partial passage of monovalent ions (Na^+^, Cl^−^, rejection 20–60%), a selectivity that permits partial demineralisation with preservation of beneficial minerals—a property of practical relevance for livestock watering applications where excessively demineralised water is also undesirable [31]. This selective transport process is illustrated schematically in Figure 2.

As shown in Figure 2, ionic and colloidal species are rejected at the membrane surface according to their charge and size, while water molecules preferentially permeate through the active layer. This size- and charge-based selectivity underlies the partial demineralisation profile described above, and explains why NF is often favoured over RO where retention of some dissolved minerals in the product water is desirable for livestock.

### 3.3. Performance Parameters Relevant to Pastoral Water Supply

For the purposes of pastoral water supply system design, the critical performance parameters are: (a) contaminant rejection efficiency, (b) permeate flux and water recovery ratio, (c) energy consumption, and (d) concentrate (brine) volume and composition. These parameters collectively determine the technological suitability of each membrane type for remote, off-grid, or semi-permanent installation conditions.

Reverse osmosis membranes generally provide high rejection of dissolved ionic contaminants, whereas nanofiltration is particularly effective for multivalent ions and hardness. Specific energy consumption is strongly dependent on feed salinity, recovery, pressure, membrane properties, and system scale. For brackish-water RO, values around 0.8–2.5 kWh/m^3^ are reported for well-optimized systems, while small or older installations may require more; NF generally operates at lower pressure and energy demand [29,33,34]. Accordingly, a single fixed energy value should not be transferred between sites without a feed-water and hydraulic design calculation.

Water recovery ratios—the fraction of feed water that emerges as treated permeate—typically range from 60 to 80% for brackish RO systems operating on low-to-moderate TDS groundwater, and from 40 to 60% for high-salinity sources [33,34]. The remainder constitutes a concentrated reject stream (brine) with TDS two to five times that of the feed, the management of which presents a specific operational and environmental challenge in arid zone contexts discussed in Section 7.

For off-grid pastoral deployment, energy demand is a primary design constraint, but PV array capacity should be calculated from the required daily water volume and the total specific electrical energy demand rather than expressed as a universal kWp-per-m^3^/day factor. Solar sizing must use site- and month-specific irradiation or PV-output data, system losses, seasonal operation, soiling, and the chosen storage strategy [14,15,35,36]. A transparent sizing method is provided in Section 7.2.

### 3.4. Fouling Mechanisms and Chemical Maintenance Strategies

Membrane fouling—the accumulation of material on or within membrane surfaces, causing flux decline or increased transmembrane pressure—is a central operational challenge in pressure-driven membrane treatment. In brackish groundwater, it may involve inorganic scaling, iron/manganese precipitates, colloids, natural organic matter, and biofilms; the relative importance of each mechanism depends on source-water chemistry and pretreatment [37,38].

Four categories should be distinguished for design and troubleshooting: (i) inorganic scaling, including CaCO_3_, CaSO_4_, BaSO_4_, and silica; (ii) colloidal fouling by clays, corrosion products, and metal hydroxide flocs; (iii) organic fouling by natural organic matter and low-molecular-weight organics; and (iv) biofouling through attachment, extracellular polymeric substances, and biofilm growth [38,39]. MF/UF are mainly affected by particulate, colloidal, organic, and biological deposition, whereas NF/RO additionally concentrate dissolved salts at the membrane surface and are therefore more sensitive to scaling and concentration polarization. Pretreatment and cleaning strategies must be selected accordingly.

Calcium carbonate scaling, the most prevalent form in hard pastoral groundwater, proceeds via:

Ca^2+^ + 2HCO_3_^−^ → CaCO_3_↓ + CO_2_ + H_2_O

and is controlled through acidification of the feed (pH adjustment to 6.0–6.5 suppresses HCO_3_^−^ and shifts the carbonate equilibrium toward CO_2_), addition of polyphosphate or polyacrylate antiscalants that inhibit crystal nucleation and growth, or both [38]. Sulphate scaling (CaSO_4_, BaSO_4_) is less responsive to pH manipulation and requires dedicated antiscalant formulations or restriction of recovery ratios to maintain the ionic product below Ksp [38].

Iron and manganese present particular pre-treatment challenges in pastoral groundwater, as Fe^2+^—thermodynamically stable in anoxic aquifer environments—oxidises to Fe(OH)_3_ upon exposure to air:

4Fe^2+^ + O_2_ + 10H_2_O → 4Fe(OH)_3_↓ + 8H^+^

The ferric hydroxide formed after oxidation can generate colloidal deposits and foul downstream membranes. Where dissolved Fe^2+^ or Mn^2+^ is significant, oxidation followed by filtration should precede NF/RO; the required oxidant dose and filtration configuration should be determined from source-water chemistry and pilot or jar tests rather than from a generic threshold [12,16].

Cleaning-in-place (CIP) restores membrane performance through chemistry selected for the dominant foulant; alkaline, acidic, oxidizing, or other manufacturer-approved cleaning steps may be required, and inappropriate cleaning can damage polyamide membranes [38]. Coagulation upstream of low-pressure membranes is well established for reducing colloidal and organic fouling [39,40]. Pre-ozonation can modify natural organic matter and may reduce some forms of organic fouling, but its net benefit is source-dependent and must be weighed against bromate risk, energy demand, and the need for additional ozone equipment [41,42]. For remote pastoral installations, simple pretreatment trains with measurable performance targets are generally preferable to chemically complex schemes unless the source-water matrix justifies them.

Published field data are currently insufficient to provide defensible Kazakhstan-specific numerical rates of membrane flux decline or universal cleaning frequencies for pastoral BWRO/NF systems. This limitation is itself a research gap: pilot monitoring should report normalized permeability, differential pressure, saturation indices, foulant composition, cleaning trigger criteria, and recovery after CIP over seasonal operation. Until such data are available, design should use conservative manufacturer limits and site-specific monitoring rather than imported fouling-rate values [38,39].

### 3.5. Operational Constraints and Suitability Assessment for Arid Pastoral Contexts

The deployment of membrane systems in remote pastoral settings introduces a series of operational constraints that do not apply to centralised urban water treatment. Chief among these are: limited technical workforce for operation and maintenance, unreliable or absent grid electricity, logistical challenges in chemical supply for pre-treatment and CIP, the absence of established brine disposal infrastructure, and the need for modular, containerised, or easily transportable system configurations [14,15].

The relative suitability of each membrane technology for pastoral application varies considerably along these dimensions. MF and UF systems, operating at low pressures and requiring minimal chemical dosing, are the most operationally straightforward and are well-established in solar-powered decentralised configurations; their principal limitation remains the inability to address mineralization. NF represents a technically compelling compromise for sources with elevated hardness and sulphate but moderate overall TDS (1000–3000 mg/L), achieving meaningful demineralisation at energy demands compatible with modestly sized PV systems. RO is indispensable for sources with TDS exceeding 3000 mg/L, though its higher energy and maintenance requirements necessitate more robust system design and operational support [29,33,34].

Table 3 presents a consolidated performance comparison of the four membrane technologies across parameters of direct relevance to pastoral water supply system design.

**Table 3:** Comparative performance characteristics of pressure-driven membrane filtration technologies (MF, UF, NF, RO) for pastoral water supply applications.

Parameter	MF	UF	NF	RO	Remarks
Pore size/MWCO	0.1–10 μm	2–100 nm 1–500 kDa	0.5–2 nm 200–1000 Da	<0.5 nm dense	Pore size governs sieving exclusion mechanism
Operating pressure (bar)	0.05–0.5	0.1–1.0	0.5–2.0	5–70 (brackish: 5–25)	Pressure demand determines energy and pump requirements
Energy consumption (kWh/m^3^)	0.05–0.3	0.1–0.5	1.0–3.0	3.0–8.0	PV-powered RO viable at 3–8 kWh/m^3^ in high-irradiance zones
TDS rejection (%)	<5	<5	30–70 (monov.) 85–98 (div.)	>95 (all)	NF selective divalent rejection suits hardness/sulphate problems
Bacterial removal (log)	3–4	6–7	6–7	>7	UF/NF/RO provide full microbial barrier
Virus removal (log)	<1	3–4	3–4	>6	MF insufficient as sole virus barrier
Cryptosporidium/Giardia	>3 log	>6 log	>6 log	>6 log	Physical size exclusion is fully effective for protozoa at MF and above
Water recovery (%)	85–98	85–95	70–85	60–80	Lower recovery increases brine volume requiring disposal
Membrane lifespan (years)	5–10	5–10	3–7	3–7	Depends on feed quality and CIP discipline
Solar-PV compatibility	Excellent	Excellent	Good	Moderate	Lower pressure = simpler PV integration; RO requires variable-frequency drive
Primary pastoral application	Pre-treatment; protozoa barrier	Standalone disinfection; pre-treatment for NF/RO	Partial demineralisation; hardness/sulphate removal	Full desalination of brackish groundwater (TDS > 3000 mg/L)	Selection governed by source TDS, microbial load, and energy availability

Sources: [29,33,34]. Values are representative engineering ranges and should be verified against the selected membrane, feed composition, recovery, pretreatment, and operating temperature.

The complete treatment train for solar-powered brackish groundwater desalination at a pastoral watering point, incorporating coarse pre-filtration, iron oxidation, MF/UF pre-filtration, antiscalant dosing, NF/RO membrane desalination, post-treatment remineralisation, and UV disinfection, is presented schematically in Figure 3. The solar PV subsystem—comprising panels, charge controller, battery bank, variable frequency drive (VFD), and high-pressure pump—is shown in parallel, illustrating the energy integration architecture that enables off-grid operation.

The treatment sequence is selected from source-water analysis rather than applied as a fixed template. A representative train comprises borehole abstraction and coarse screening; aeration/oxidation with Fe/Mn filtration where required; MF/UF pretreatment; antiscalant or pH conditioning before NF/RO; post-membrane remineralisation; terminal UV disinfection; and covered storage or direct distribution. NF or RO is included only when dissolved-solids removal is needed. Remineralisation after RO is important because low-alkalinity permeate can be chemically aggressive and poorly buffered; CO_2_/calcite contactors can restore calcium, alkalinity, and pH stability, thereby reducing corrosion risk in downstream infrastructure [10,12]. For livestock supply, the remineralisation target should be set to meet species-appropriate water-quality requirements and palatability objectives rather than to maximize hardness [22,23,43,44]. Concentrate management is addressed separately in Section 7.3.

## 4. Ultraviolet Disinfection

### 4.1. Physical and Photochemical Mechanism of Inactivation

Ultraviolet (UV) disinfection exploits the germicidal properties of electromagnetic radiation in the wavelength range of 200–300 nm to inactivate pathogenic microorganisms without the addition of chemical reagents. The primary mechanism of microbial inactivation is photochemical damage to nucleic acids—both deoxyribonucleic acid (DNA) and ribonucleic acid (RNA)—upon direct absorption of UV-C photons [45]. The UV action spectrum for DNA damage peaks at approximately 260 nm, corresponding closely to the absorption maximum of the pyrimidine bases thymine (T) and cytosine (C), which are the principal chromophores in DNA at germicidal wavelengths [46].

Low-pressure (LP) mercury vapour lamps, the most widely deployed UV source in water treatment practice, emit monochromatic radiation predominantly at 254 nm—sufficiently close to the DNA absorption maximum to achieve efficient energy transfer to the nucleic acid target. Medium-pressure (MP) mercury lamps emit polychromatic radiation across a broader spectrum (200–320 nm), offering advantages in terms of reduced photoreactivation potential and broader action against a wider range of molecular targets, at the cost of higher energy consumption [46].

Pastoral water sources that are exposed to faecal contamination may contain *E. coli* and other enteric bacteria as well as protozoan pathogens such as Cryptosporidium and Giardia [10,26]. These protozoa are comparatively resistant to conventional chlorination at practicable drinking-water doses but are highly susceptible to appropriately delivered UV fluence [13,45]. UV-C photons induce photochemical lesions in nucleic acids, including cyclobutane pyrimidine dimers, which block replication and render microorganisms non-infective [46,47].

Low-pressure (LP) mercury vapour lamps emit monochromatic radiation at 254 nm and represent the most cost-effective UV source for pastoral water treatment. Medium-pressure (MP) lamps offer broader spectral output and reduced photoreactivation potential, at a higher energy cost [46]. UV-LED systems, while more expensive per unit output than conventional mercury lamps, offer additional advantages for remote pastoral deployment: instant start-up, longer operational lifespan (50,000+ h), and mercury-free construction [48]. The underlying inactivation mechanism is illustrated in Figure 4.

As illustrated in Figure 4, UV-C photons are absorbed directly by microbial nucleic acids, producing photoproducts—principally pyrimidine dimers—that block replication and transcription. Because this mechanism does not depend on a chemical residual, inactivation efficacy is governed primarily by the delivered UV dose rather than by contact time with a disinfectant, distinguishing UV disinfection from the chlorination-based approaches discussed in Section 4.3.

### 4.2. Inactivation Kinetics and Dose–Response Relationships

The applied UV fluence F (mJ/cm^2^) is the product of irradiance E (mW/cm^2^) and exposure time t (s): F = E·t. Required fluence depends on organism, reactor hydraulics, UV transmittance, and the validation method. Collimated-beam studies show high UV sensitivity of Giardia and Cryptosporidium, with multi-log inactivation at relatively low fluence [13,47,49]. Practical full-scale design, however, should be based on validated reactor reduction-equivalent dose rather than a universal ‘minimum’ value. Enteric bacteria are generally more UV-sensitive than adenovirus, which can require substantially higher doses for equivalent log reduction [13,46]. Table 4 summarises representative literature values and should be interpreted as comparative laboratory data, not as a substitute for reactor validation.

### 4.3. Advantages of UV Disinfection over Chlorination for Pastoral Applications

The selection of a disinfection technology for pastoral water supply is conditioned not only by microbiological efficacy but also by operational, chemical, and acceptability considerations specific to remote and low-resource deployment contexts. Compared to chlorine-based disinfection—the most widely applied method globally—UV disinfection presents several important advantages relevant to these conditions.

Most critically, UV treatment produces no disinfection by-products (DBPs) of the type generated by chlorination, which forms regulated carcinogens (trihalomethanes and haloacetic acids) upon reaction with natural organic matter [50]. UV efficacy is also independent of pH, unlike chlorine, whose germicidal activity depends on the HOCl/OCl^−^ equilibrium (pKa 7.5) and is therefore reduced in the alkaline groundwaters typical of arid-zone aquifers (pH 7.5–8.5) [50].

The principal limitation of UV disinfection, which must be addressed in system design, is the absence of residual disinfection capacity. Unlike chlorine, which maintains a residual concentration capable of controlling microbial regrowth in distribution pipes and storage tanks, UV treatment provides no continuing protection after the water leaves the irradiation chamber [10,12]. In pastoral water supply systems where treated water is stored in open or partially open tanks prior to delivery to watering troughs, recontamination pathways can negate the disinfection achieved in the UV reactor. This limitation is typically addressed by positioning UV reactors immediately upstream of the point of use, by designing closed distribution systems that minimize recontamination opportunities, or by combining UV treatment with a low residual chlorine dose sufficient to suppress regrowth without reaching THM-forming concentrations. Where treated water is stored in open or semi-open tanks prior to delivery to watering troughs—as is common at pastoral installations—a low residual chlorine dose (0.2–0.5 mg/L free chlorine) should be applied post-UV to suppress microbial regrowth during storage; without this secondary barrier, the disinfection achieved in the UV reactor cannot be maintained through to the point of animal consumption [10,12,50].

### 4.4. Solar UV Disinfection (SODIS) as a Low-Technology Option for Remote Pastoral Points

Solar water disinfection (SODIS)—exposing water in clear PET bottles to solar UV-A and thermal heating—achieves 3–4 log reduction in common enteric pathogens after several hours of direct sunlight, and is WHO-recommended for household-scale use [51]. Its relevance to pastoral watering points, however, is limited to small supplementary volumes rather than the throughputs (10–25 m^3^/day) addressed by the membrane-based systems that are this review’s focus; it is noted here only as a low-cost fallback option at the smallest, most remote sites.

### 4.5. Turbidity Dependence and Pre-Treatment Requirements

The efficacy of UV disinfection is directly dependent on the optical transmittance (UVT) of the water to be treated. Suspended particles, dissolved organic matter, iron, and other UV-absorbing substances attenuate the UV radiation field within the reactor, reducing the effective fluence delivered to target organisms relative to the applied lamp output. The relationship between UV absorbance (UVA_254_, measured at 254 nm in cm^−1^), UVT, and effective fluence is expressed as:

UVT (%) = 10^(−UVA254 × path length)^ × 100

UV performance depends strongly on water optical quality. Low UV transmittance caused by suspended solids, dissolved organic matter, iron, or manganese reduces fluence delivery and can shield microorganisms. No single UVT threshold is universally applicable to every validated reactor; the allowable UVT range must be defined by the reactor’s validation envelope and target log reduction [10,48]. Where source water has elevated turbidity or oxidizable iron/manganese, oxidation–filtration or MF/UF pretreatment is therefore appropriate before UV treatment.

It should be noted that the UVT requirement for pastoral water supply systems treating water for livestock use may be less stringent than for human drinking water, given the greater microbiological tolerance of ruminants to sub-optimal disinfection. This distinction has design implications: in systems serving solely livestock watering points, simplified pre-filtration may be sufficient to achieve the UVT levels required for a 2-log rather than 4-log pathogen reduction target, reducing capital and operational costs accordingly.

### 4.6. Integration of UV Disinfection Within Multi-Barrier Treatment Systems

In the context of pastoral water supply in arid zones, UV disinfection is most effectively deployed not as a standalone treatment technology but as a disinfection polishing step within an integrated multi-barrier system. The logical position of a UV reactor within a treatment train depends on the upstream processes employed and the quality of water presented to the UV stage.

In a system incorporating MF or UF pre-filtration followed by NF or RO desalination, the UV reactor is appropriately positioned downstream of the membrane stage, where turbidity and UV-absorbing constituents are minimized. The membrane stage provides an independent physical barrier, while terminal UV supplies a complementary disinfection barrier [28,48]. This arrangement is consistent with the multi-barrier risk-management approach described in WHO drinking-water guidance [10].

Where membrane desalination is not required—for example, in sources with acceptable TDS but significant microbiological contamination—standalone UF combined with UV polishing provides a compact, chemical-free treatment train with full bacterial, viral, and protozoan removal capability. This configuration is particularly relevant to pastoral watering points in sub-humid zones where groundwater mineralization is not a primary concern, but seasonal surface contamination of shallow aquifers creates periodic microbiological risk. A comparative assessment of UV disinfection, ozonation, and chlorination for pastoral water supply systems is presented in Table 5.

## 5. Ozonation

### 5.1. Chemistry of Ozone and Its Decomposition in Water

Ozone (O_3_) is a powerful oxidising agent, the third strongest after fluorine and the hydroxyl radical (E° = +2.07 V). It acts via direct, selective oxidation by molecular O_3_, and indirect, non-selective oxidation via hydroxyl radicals (•OH, E° = +2.80 V) generated as O_3_ decomposes in water [53,54]. Ozone decomposition and •OH yield increase with pH and temperature—advantageous for micropollutant degradation, but this same effect accelerates bromate formation in bromide-containing waters, a key trade-off for treatment design in Central Asian pastoral groundwater [54]. Ozone half-life in clean water falls from roughly 20–30 min at 15 °C to 5–10 min at 25 °C at neutral pH, and is considerably shorter in high-NOM or high-alkalinity waters [53,54]. The two parallel oxidation pathways are summarised in Figure 5.

As shown in Figure 5, ozone reacts with contaminants through two parallel pathways: direct, selective attack by molecular O_3_, and indirect, non-selective oxidation by hydroxyl radicals generated during ozone decomposition. The relative contribution of each pathway governs both the rate of micropollutant removal and the extent of bromate formation described above, and therefore shapes the water-quality trade-offs associated with ozonation in pastoral applications.

### 5.2. Oxidation of Inorganic Contaminants Relevant to Pastoral Water Supply

Ozone rapidly oxidises Fe^2+^ to filterable Fe(OH)_3_ (stoichiometric demand 0.43 mg O_3_/mg Fe^2+^) and Mn^2+^ to MnO_2_ (0.88 mg O_3_/mg Mn^2+^), and oxidises H_2_S to sulphate, eliminating odour [55]. However, ozone-based oxidation of these species carries a disproportionate capital and energy cost—and a bromate formation risk in bromide-containing source waters—relative to conventional aeration-filtration, which achieves equivalent Fe^2+^/Mn^2+^ removal and H_2_S stripping at a fraction of the cost [54,55]. For pastoral deployment where ozone is not already required for disinfection or micropollutant control, aeration combined with dual-media filtration is therefore the preferred pre-treatment strategy.

### 5.3. Disinfection Efficacy and CT Concept

Ozone disinfection performance is expressed using the CT concept, the product of residual ozone concentration C (mg/L) and effective contact time T (min). CT requirements are strongly temperature-dependent. For Giardia, U.S. EPA CT tables for ozone at pH 6–9 give 0.72 mg·min/L for 3-log inactivation at 20 °C and 1.90 mg·min/L at 5 °C [52]. Temperature and pH should therefore be reported whenever a Giardia ozone CT value is quoted.

*Cryptosporidium parvum* is more resistant to ozone than Giardia. The U.S. EPA LT2 toolbox gives a 3-log Cryptosporidium ozone CT of 12 mg·min/L at 20 °C, increasing to 19 mg·min/L at 15 °C and 47 mg·min/L at 5 °C [52]. The regulatory table is indexed by temperature; pH and source-water chemistry should nevertheless be reported because they influence ozone decay and bromate chemistry [53,54]. These values support retaining UV as a complementary terminal barrier where robust Cryptosporidium control is required.

Table 6 summarises the second-order rate constants for key reactions of molecular ozone and hydroxyl radical with contaminants relevant to pastoral water supply, together with indicative CT values for pathogen inactivation.

### 5.4. Disinfection By-Products of Ozonation: Bromate Formation and Control

The principal regulated disinfection by-product of ozonation is bromate (BrO_3_^−^), formed through oxidation of naturally occurring bromide (Br^−^) by pathways involving molecular ozone and radical intermediates. The WHO drinking-water guideline value is 10 μg/L, and bromate formation is an important carcinogenicity concern in ozone process design [10,54]. Bromate formation proceeds through a multi-step network that can be represented schematically as:

Br^−^ → HOBr/OBr^−^ → BrO• → BrO_2_^−^ → BrO_3_^−^

The conversion of bromide to bromate proceeds through multiple molecular-ozone and radical steps; the relative importance of those pathways depends on pH, bromide, ozone exposure, dissolved organic matter, alkalinity, and temperature [53,54]. For this reason, the simplified reaction sequence above should be read as a pathway summary rather than a complete elementary mechanism.

For Kazakhstan and Central Asia, bromide should be treated as a source-characterization parameter rather than assumed from regional geology. Published bromide datasets for pastoral aquifers are too sparse to justify a universal concentration range. Before ozonation is selected, bromide should be measured together with pH, alkalinity, dissolved organic carbon, temperature, and ozone demand. Where bromate formation potential is significant, mitigation can include pH adjustment, ammonia-based pathways where appropriate, ozone-dose/contact-time optimization, or carefully controlled peroxone operation, validated for the actual source water [54].

Secondary ozonation by-products of relevance include aldehydes (formaldehyde HCHO, acetaldehyde CH_3_CHO, glyoxal (CHO)_2_) and carboxylic acids generated by the partial oxidation of NOM. While not acutely toxic at typical formation concentrations, these compounds increase the biodegradable dissolved organic carbon (BDOC) content of the water, stimulating microbial regrowth in post-ozonation distribution—a concern addressed by incorporating biological activated carbon (BAC) filtration downstream of the ozone stage to consume BDOC before distribution [53,62].

### 5.5. Ozone Generation and Energy Considerations for Remote Pastoral Deployment

Ozone is generated on site, commonly by corona discharge from dry air or oxygen. Generator electricity demand is often expressed per kilogram of ozone produced; values of roughly 7–20 kWh/kg O_3_ are reported depending on feed gas and equipment [61]. At an applied dose of 3 mg O_3_/L, this corresponds to approximately 0.021–0.060 kWh/m^3^ for ozone generation itself (3 g O_3_/m^3^ × 7–20 kWh/kg), before oxygen preparation, pumps, cooling, off-gas destruction, and other ancillary loads are included. Reporting ozone energy per cubic metre should therefore distinguish the generator load from the total subsystem load.

However, ozone generation also requires ancillary equipment—an air preparation or oxygen supply system, a contact reactor with adequate gas–liquid mass transfer (typically a bubble diffuser or venturi injector column), an off-gas ozone destructor (to prevent atmospheric release of residual O_3_, which is toxic at concentrations above 0.1 ppm), and process instrumentation for ozone residual monitoring. The capital cost and operational complexity of this equipment base substantially exceeds that of UV systems, making ozonation more appropriate for semi-permanent or permanent pastoral water supply installations treating larger water volumes (>1 m^3^/h) than for small, fully mobile units [59,61].

Henry’s law data must be reported with an explicit convention because reciprocal forms use different units. Using the solubility convention Hs(cp) = c/p, a representative value for CO_2_ in water near 25 °C is approximately 3.4 × 10^−2^ M·atm^−1^, corresponding to an inverse constant of about 29 atm·L·mol^−1^; representative ozone values are lower and temperature-dependent [63]. The previous comparison of CO_2_ as ~30 M·atm^−1^ mixed the reciprocal conventions and has therefore been corrected. In practice, ozone contactors still require efficient gas–liquid mass transfer because dissolved ozone is reactive and decays in the treated water.

### 5.6. Ozone-Based Advanced Oxidation Processes (O_3_/H_2_O_2_ and O_3_/UV) and Membrane Integration

To avoid duplication, the mechanisms and design implications of O_3_/H_2_O_2_ (peroxone), O_3_/UV, UV/H_2_O_2_, and their integration with membrane treatment are consolidated in Section 6.3, and Section 5 therefore focuses on standalone ozonation chemistry, disinfection, by-products, generation, and field constraints.

### 5.7. Applicability and Constraints of Ozonation in Arid Pastoral Contexts

Ozonation offers a compelling set of capabilities for pastoral water treatment in arid zones: efficient removal of iron, manganese, and colour without chemical sludge generation; powerful disinfection of bacteria and Giardia at low CT values; reduction in membrane fouling in integrated treatment trains; and the capacity for micropollutant removal through AOP configurations where wastewater-affected sources are used. These properties make ozonation particularly attractive as a component of integrated treatment systems at semi-permanent pastoral infrastructure—seasonal grazing stations, veterinary facilities, and rural service settlements—where system complexity and energy supply are less constraining than at remote single-point watering sites [42,53,55].

The main constraints on ozonation in remote pastoral systems are the need for on-site electrical generation, additional gas-transfer and off-gas equipment, the absence of a persistent disinfectant residual, and bromate risk where bromide is present. Ozone decay also means that treated water stored or distributed after the contact tank can be recontaminated unless the network is closed or an appropriate residual-disinfection strategy is used [53,54,55]. For low-volume remote sites, UV is usually simpler to operate. Ozone is better reserved for cases in which its oxidative functions—such as Fe/Mn oxidation, colour removal, or micropollutant treatment—justify the added complexity.

## 6. Hybrid and Combined Treatment Systems

### 6.1. Rationale for Multi-Barrier Treatment Approaches

No single water treatment technology is capable of simultaneously addressing the full spectrum of contaminants typically present in groundwater sources of arid pastoral zones. Brackish groundwater in such regions commonly exhibits elevated total dissolved solids (TDS), turbidity, microbiological contamination, and organic micropollutants—a combination that exceeds the removal capacity of any individual process. The multi-barrier concept, endorsed by the World Health Organization within its Water Safety Plan framework, posits that the sequential application of complementary treatment steps provides redundancy and cumulative contaminant removal, thereby ensuring consistent compliance with drinking water and livestock watering standards even under variable source water conditions [10].

In pastoral settings characterized by remoteness, limited operator expertise, and intermittent energy supply, multi-barrier systems should emphasize mechanical robustness, minimal chemical handling, and maintainability. Modular treatment units can provide redundancy while allowing individual components to be serviced without redesigning the complete train [10,12,64].

From the perspective of pastoral water supply, a multi-barrier approach also provides resilience to spatial and seasonal variability in source-water quality. Kazakhstan groundwater studies document substantial differences in mineralization and hydrochemistry among regions, aquifers, and individual wells [9,11,17,20]. Treatment trains should therefore be configured from measured source-water conditions and monitored over time rather than assuming a static regional composition.

### 6.2. Coagulation–Flocculation as Pre-Treatment for Membrane Systems

One of the most critical preparatory steps preceding membrane filtration is the reduction in suspended solids, colloidal particles, and natural organic matter (NOM) through coagulation–flocculation. Without effective pre-treatment, membrane surfaces are rapidly fouled, leading to flux decline, increased transmembrane pressure (TMP), and shortened membrane lifespan—all of which are particularly costly to remediate in remote environments. Malkoske et al. [39] conducted a comprehensive review of coagulation/flocculation pretreatment configurations and demonstrated that optimized coagulation significantly extends inter-cleaning intervals and reduces irreversible fouling in both microfiltration (MF) and ultrafiltration (UF) systems.

The most widely applied coagulants are aluminum sulfate (alum, Al_2_(SO_4_)_3_) and ferric chloride (FeCl_3_). The coagulation mechanism proceeds via two primary pathways: charge neutralisation and sweep flocculation. Upon dissolution, Al^3+^ and Fe^3+^ ions hydrolyse to form positively charged hydroxo-complexes, which precipitate as amorphous flocs:

Al^3+^ + 3H_2_O → Al(OH)_3_↓ + 3H^+^

Fe^3+^ + 3H_2_O → Fe(OH)_3_↓ + 3H^+^

These hydroxide precipitates adsorb colloidal particles and NOM, which are subsequently removed by sedimentation or media filtration prior to membrane contact. Optimal coagulant dose and pH range (typically pH 6.5–7.5 for alum; pH 5.0–8.0 for FeCl_3_) must be determined by jar testing for each specific source water [39,40].

For pastoral water treatment, pre-hydrolysed coagulants such as polyaluminium chloride may offer operational advantages over conventional alum in some waters, but coagulant selection and dose must be confirmed by jar testing. A 2025 review by Łukasiewicz [65] summarizes coagulation–sedimentation for natural organic matter and emerging contaminants and supports its role as a pretreatment step rather than a universal standalone solution. Natural coagulants, including Moringa oleifera-derived materials, are also under investigation, although standardized performance and residual-organic effects must be considered [66].

### 6.3. UV/O_3_ Advanced Oxidation Processes (AOPs)

When source water contains refractory organic micropollutants, advanced oxidation processes (AOPs) can be considered because they generate highly reactive hydroxyl radicals (•OH). Their use should be source-driven: field evidence for pastoral groundwater treatment in Central Asia remains limited, so O_3_/UV, O_3_/H_2_O_2_, or UV/H_2_O_2_ should not be included routinely unless micropollutants or other specific treatment objectives justify the added energy and complexity [42,58,67].

The UV/H_2_O_2_ system, one of the most widely implemented AOPs, generates OH via photolytic cleavage of hydrogen peroxide:

H_2_O_2_ + hν (UV-C, 254 nm) → 2**•**OH

The O_3_/UV combination further enhances **•**OH production through ozone photolysis, as described by the following reaction sequence:

O_3_ + H_2_O + hν → H_2_O_2_ + O_2_

H_2_O_2_ + hν → 2**•**OH

The O_3_/H_2_O_2_ (peroxone) process increases radical production by accelerating ozone decomposition. A commonly used shorthand depicts the reaction of HO_2_^−^ with O_3_ and the formation of radical species, including superoxide (O_2_•^−^), followed by additional reactions that generate **•**OH. Mechanistically, however, the pathway is more complex: formation and decomposition of a peroxone adduct can yield HO_2_•/O_2_•^−^ and O_3_•^−^, and subsequent O_3_•^−^ chemistry produces **•**OH [68,69]. Consequently, the reaction sequence should be interpreted as a radical chain mechanism rather than a single elementary step. H_2_O_2_:O_3_ ratio, pH, alkalinity, and radical scavengers must be optimized experimentally for the actual water matrix.

A practical constraint of ozone-based AOPs is bromate formation when bromide is present. Because bromide occurrence and bromate yield are source-specific, Kazakhstan groundwater should be characterized before O_3_/UV or peroxone is selected. Mitigation strategies include pH adjustment, ammonia addition where appropriate, optimized ozone exposure, and carefully controlled H_2_O_2_ dosing [54,55].

Hübner et al. [67] emphasize that AOP research should evaluate not only parent-compound removal but also transformation products, energy demand, and matrix effects. This is particularly important when treated water is intended for livestock or human use, because incomplete oxidation can replace one set of compounds with another rather than achieve complete mineralization.

### 6.4. Solar-Powered Integrated Systems: PV-Driven RO + UV Disinfection

The convergence of declining photovoltaic (PV) module costs, improved battery storage technologies, and modular water treatment hardware has made solar-powered integrated treatment systems increasingly viable for off-grid pastoral applications. A typical system architecture for arid pastoral conditions couples a PV array with a variable-frequency-driven (VFD) brackish water reverse osmosis (BWRO) unit and a UV disinfection reactor in a single containerised or skid-mounted configuration that can be deployed at remote watering points without grid connection [14,15].

PV-driven membrane systems are attractive where solar availability and water demand coincide, and water storage can often substitute for part of the electrical battery capacity. Field testing in remote Australia demonstrated PV-powered UF + RO/NF treatment of brackish bore water, with specific energy consumption of 2–8 kWh/m^3^ depending on salinity and operating conditions [70]. More recent reviews show continued improvement in solar-RO integration, but performance remains strongly dependent on scale, membrane design, intermittency management, and storage strategy [14,15,71].

The integration of UV disinfection as a polishing step downstream of RO adds minimal additional energy demand (typically 0.02–0.05 kWh/m^3^) while providing a critical safeguard against post-membrane microbial re-contamination during distribution to multiple watering points. This is particularly important in pastoral settings where distribution lines may extend several kilometres from the treatment unit to distant paddock troughs. The WHO Water Safety Plan framework explicitly recommends multi-barrier treatment including a terminal disinfection step for all rural water supplies distributed through open or semi-open networks [45,48].

Renewable-energy desalination can reduce dependence on delivered fuel at off-grid sites, but no universal distance threshold makes solar automatically cheaper than diesel. Site-level comparison should include PV and generator CAPEX, fuel price and transport, operating hours, replacement schedules, battery or water-storage needs, and discount rate [14,15,34,35,36,71]. Section 7.2 therefore provides a transparent sizing and break-even formulation rather than retaining the unsupported ‘50 km’ rule. The physical arrangement of these components in a representative off-grid installation is shown in Figure 6.

Figure 6 situates the individual components discussed in this section within a single installation: the solar PV array and power-conditioning unit supply electrical power to the water treatment train, while treated water passes to covered storage before gravity or pumped delivery to the livestock trough. Separating the water-flow, power-flow, and control-signal paths in this way clarifies the points at which the system depends on solar availability, and where battery or storage buffering is required to sustain operation after sunset.

## 7. Technical, Economic, and Environmental Aspects of Implementation

The deployment of advanced water treatment systems in arid pastoral zones is governed not only by technical performance criteria but also by economic feasibility, energy availability, environmental risk, and local maintenance capacity. Unlike urban water infrastructure, pastoral water supply systems operate at small to medium scale, often without grid electricity, qualified operators, or proximity to spare-parts supply chains. An integrated assessment of capital and operational costs, energy requirements, concentrate management, and environmental footprint is therefore essential for identifying appropriate technology combinations and for designing cost-effective, sustainable systems suited to the specific conditions of Kazakhstan’s and Central Asia’s pastoral regions [2,10].

### 7.1. Capital and Operational Costs (CAPEX/OPEX) Across Technologies

The economic viability of a water treatment technology for pastoral applications is typically expressed as the total cost per cubic metre of treated water, decomposed into capital expenditure (CAPEX)—covering equipment procurement, civil works, and commissioning—and operational expenditure (OPEX)—covering energy, chemicals, membrane replacement, labour, and maintenance. For small-scale decentralised systems (production capacity 5–100 m^3^/day), unit costs are substantially higher than for large municipal plants due to the absence of economies of scale, higher specific energy consumption at low flow rates, and elevated per-unit logistics costs [14,15,34,64].

Published cost ranges for small decentralized treatment systems vary widely with capacity, feed-water quality, local labour, energy source, pretreatment, and procurement. UV generally has low operating energy demand, while ozone requires additional generation and gas-handling equipment. For desalination, BWRO operating cost is strongly influenced by specific energy consumption and membrane replacement [33,34]. The values in Table 7 are therefore retained only as indicative planning ranges and should not be interpreted as vendor quotations or universally transferable costs.

For solar-powered BWRO + UV systems, total water cost depends on both treatment equipment and the energy subsystem. The comparison in Table 7 separates electrical demand from the source of electricity: PV supply does not make energy consumption zero. Project appraisal should calculate lifecycle cost using site-specific solar data, delivered fuel cost, financing assumptions, and maintenance logistics [14,15,34,35,36,71].

Table 7 is intended as a screening comparison rather than a deterministic cost model. UV and UF can be economical where their treatment functions are sufficient, whereas BWRO is justified when dissolved-solids removal is required. Solar integration can reduce exposure to recurring fuel costs but adds PV, controls, and—where used—battery capital. Ozonation should be selected only where its additional oxidative functions provide measurable value. Final technology selection should therefore be based on source-water quality and lifecycle cost, not on nominal CAPEX or a single literature OPEX range.

### 7.2. Energy Consumption and Solar Integration Feasibility

Energy consumption is a major component of BWRO operating costs. For brackish water treatment, optimized RO systems can operate at specific energy consumptions of approximately 0.8–2.5 kWh/m^3^, although smaller-scale or less optimized installations may require higher values [33]. UV polishing typically contributes only a minor additional electrical load, whereas the energy demand of ozonation depends on the applied ozone dose and the associated ancillary equipment. These values should therefore be incorporated into an explicit site-specific energy balance rather than used to size the photovoltaic system directly from nominal plant capacity.

A transparent first-pass PV sizing approach can be based on the daily electrical energy demand:

*E_d_* = *Q* × *SEC_total_*

where *E_d_* is the daily electrical energy demand (kWh/day), *Q* is the treated-water production rate (m^3^/day), and *SEC_total_* is the total specific electrical energy consumption of the treatment system (kWh/m^3^). The required photovoltaic capacity can then be approximated as:

$$P_{\mathrm{PV}}=\;\frac{E_{d}}{H_{\mathrm{PS}}\times \mathrm{PR}\;}$$

where *P_PV_* is the required PV capacity (kWp), *H_PS_* is the site- and month-specific equivalent peak-sun-hours (h/day), and *PR* is the overall system performance ratio accounting for temperature effects, inverter and wiring losses, module mismatch, and soiling [34,35,36]. For illustration only, *Q* = 15 m^3^/day and *SEC_total_* = 1.7 kWh/m^3^ give *E_d_* ≈ 25.5 kWh/day; with *H_PS_* = 5 h/day and *PR* = 0.75, *P_PV_* ≈ 6.8 kWp. The same load at 3 h/day requires ≈ 11.3 kWp. Monthly site data, not annual averages, should govern the design month.

#### PV Sizing, Storage, Soiling, and Solar–Diesel Cost Comparison

Solar-resource inputs for preliminary system sizing should be obtained from an established regional or global dataset, such as the Global Solar Atlas, and complemented by site-specific measurements or validated project-grade solar-resource data where detailed investment-grade design is required [35]. In dusty environments, soiling can materially reduce photovoltaic output; therefore, the design performance ratio should include a site-specific soiling allowance together with an appropriate panel-cleaning schedule [36]. Where water demand can be shifted to daylight hours, treated-water storage may be more practical and less costly than extensive battery storage.

When battery storage is required, a first-order estimate of usable battery capacity can be expressed as:

$$C_{\mathrm{bat}}=\frac{E_{\mathrm{night}}N_{\mathrm{aut}}}{\mathrm{DoD}\;\times \;\eta_{\mathrm{bat}}}$$

where *C_bat_* is the required nominal battery capacity (kWh), *E_night_* is the load to be served outside PV production, *N_aut_* is the required autonomy, *DoD* is allowable depth of discharge, and *η_bat_* is battery round-trip efficiency.

For a diesel-powered system, the fuel-related treatment cost can be approximated as:

$$C_{\mathrm{diesel},\mathrm{fuel}}=\;{\frac{{\mathrm{SEC}}_{\mathrm{total}}P_{\mathrm{fuel},\mathrm{del}}}{\eta_{\mathrm{gen}}{\mathrm{LHV}}_{\mathrm{diesel}}}\;}_{.}$$

where *SEC_total_* is the total electrical energy requirement of the treatment system (kWh/m^3^), *P_fuel_,_del_* is the delivered diesel price (USD/L), *η_gen_* is the electrical efficiency of the diesel generator, and *LHV_diesel_* is the lower heating value of diesel expressed in kWh/L. If the delivered fuel price is represented as *P_fuel_,_del_* = *P*_0_ + *αd*, where *α* is a distance-related transport surcharge and *d* is delivery distance, the effect of remoteness on diesel operating cost can be evaluated explicitly.

A complete solar–diesel comparison should be based on a project-specific discounted cash-flow analysis over the selected project lifetime and should include PV and battery capital costs, component replacement, O and M, diesel-generator capital and maintenance costs, fuel consumption, fuel transport, financing, and system utilization. Consequently, no universal break-even distance can be assigned to remote pastoral installations.

As an illustrative calculation only, consider a treatment system with *SEC_total_* = 1.7 kWh/m^3^, a diesel lower heating value of 9.8 kWh/L, and a generator electrical efficiency of 0.30. The fuel-only diesel contribution is approximately USD 0.58/m^3^ at a delivered diesel price of USD 1.00/L and USD 0.87/m^3^ at USD 1.50/L. If an annualized PV-plus-storage cost of USD 0.75/m^3^ is assumed for illustration, equality with the diesel fuel-only contribution occurs at a delivered diesel price of approximately USD 1.30/L. Under the hypothetical logistics relation *P_fuel,del_* = 1.00 + 0.01d (USD/L, with d in km), this corresponds to approximately 30 km. This value is not a deployment threshold; it only demonstrates the calculation procedure. Actual break-even conditions must be determined from local fuel prices, transport costs, generator loading, PV and battery costs, financing conditions, component replacement schedules, O&M requirements, and system utilization [14,15,36,71].

### 7.3. Brine and Concentrate Management: Environmental Risks and Disposal Strategies in Arid Zones

Reverse osmosis and nanofiltration generate a concentrated reject stream (brine or concentrate) containing elevated concentrations of TDS, hardness ions (Ca^2+^, Mg^2+^), sulphates (SO_4_^2−^), and, in some source waters, heavy metals and trace contaminants. For a BWRO system operating at a typical recovery ratio of 60–75%, the concentrate volume represents 25–40% of the feed flow at TDS levels two to four times that of the source water. In the pastoral context of arid Kazakhstan, where groundwater TDS frequently exceeds 2000–5000 mg/L, concentrate TDS may reach 8000–20,000 mg/L—a significant disposal challenge [72].

Common concentrate-management options include evaporation ponds, regulated surface discharge where environmentally permissible, deep-well injection in suitable geological settings, and further concentration or resource recovery [72,73]. Evaporation ponds can be attractive in arid regions but require land, engineered lining, leak monitoring, and wildlife-risk management. Deep-well injection requires appropriate confining geology, well integrity, regulatory approval, and long-term monitoring; it should not be presented as a generic low-risk option [73].

Zero-liquid-discharge (ZLD) approaches further concentrate brine to minimize liquid discharge, but thermal or membrane-concentration stages can impose substantial energy and capital penalties. They are therefore difficult to justify for small off-grid pastoral systems unless environmental constraints are unusually strict [72,73,74]. Brine valorisation may recover industrial salts or other products, but economic feasibility depends on concentration, purity, market access, and the cost of separation [74].

For pastoral systems in Kazakhstan, concentrate management should be selected through site-specific hydrogeological and environmental assessment. Practical priorities are to avoid unnecessary over-recovery that promotes scaling, minimize concentrate volume within membrane constraints, use lined evaporation or other controlled disposal where appropriate, and prevent seepage to shallow groundwater [73]. Direct use of recovered salts as livestock mineral supplements should not be assumed: RO concentrate can enrich undesirable ions and trace contaminants, so any feed-related use would require full compositional analysis and compliance with feed-safety regulations.

### 7.4. Maintenance Requirements and Local Capacity Considerations

Long-term sustainability of remote treatment systems depends on access to maintenance, spare parts, consumables, and appropriately trained operators. Membrane and lamp replacement intervals are not fixed properties: they depend on feed quality, pretreatment, operating hours, cleaning practice, and manufacturer specifications. Lifecycle costing should therefore use documented replacement assumptions and sensitivity analysis rather than universal service-life values [33,38,65].

Remote telemetry can reduce unnecessary site visits by tracking feed pressure, permeate conductivity/TDS, flow, tank level, UV intensity, and alarm states. Packaged skid-mounted systems can simplify maintenance, but the training requirement should be defined from the actual equipment, chemical hazards, and local staffing model; a universal two- or three-day training period is not evidence-based. Operating manuals, lockout procedures, chemical handling, troubleshooting, and spare-parts planning should form part of commissioning.

Chemical cleaning (CIP—Clean-In-Place) of membranes requires periodic use of citric acid (for carbonate/sulphate scale), sodium hydroxide (for organic fouling), and sodium hypochlorite (for biofouling). These chemicals must be stored safely on-site, requiring appropriate containment and handling protocols. In the absence of qualified chemical technicians, simplified cleaning protocols with pre-formulated cleaning solutions should be standardised and provided with each system. Antiscalant dosing systems, which reduce the frequency of CIP cleaning by suppressing scale formation at the membrane surface, are strongly recommended for Kazakhstan’s hard, sulphate-rich groundwater [37,38].

### 7.5. Life Cycle Assessment (LCA) Perspectives on Ecological Footprint

LCA is increasingly adopted to support sustainable decision-making and environmental performance assessment of rural water infrastructure projects and is frequently recommended by international funding organizations during project appraisal [75,76].

A 2024 comparative LCA of four municipal disinfection methods [77] found low-pressure UV to have the lowest operational environmental impact among the compared disinfection options, whereas ozone and UV-LED were more electricity-intensive. The study also showed that electricity mix materially influences life-cycle results. This supports a cautious conclusion for pastoral systems: renewable electricity can improve the environmental profile of electrically driven treatment, but the magnitude of improvement must be calculated for the actual PV system, storage configuration, and treatment train rather than transferred as a fixed percentage [76,77,78].

Tran and Weidhaas [78] used LCA to compare multiple industrial wastewater treatment trains containing technologies such as ion exchange, RO, UV/H_2_O_2_, and ozone. Although the application is different from pastoral groundwater, the study illustrates that environmental ranking is sensitive to process configuration, replacement assumptions, and operating inputs. For the present review, it is therefore more defensible to recommend site-specific LCA than to label BWRO + UV universally ‘environmentally optimal’.

A comprehensive environmental assessment of water treatment technologies for pastoral contexts in Kazakhstan should incorporate, at minimum, the following impact categories: GWP, water consumption (accounting for concentrate losses), land use (for evaporation ponds), and ecotoxicity potential of chemical inputs (antiscalants, cleaning agents). Where data permit, a social LCA dimension addressing operator safety and community health co-benefits should also be incorporated to support evidence-based policy decisions at the national level [76,77,78,79,80].

## 8. Agricultural Benefits and Pastureland Sustainability

Access to adequate quantities of acceptable-quality water is a foundational determinant of livestock performance in arid and semi-arid pastoral systems. High salinity, sulphate, nitrate, microbial contamination, or poor palatability can reduce voluntary intake or impair animal performance, but responses depend strongly on species, age, diet, climate, and acclimation [81]. Kazakhstan groundwater mineralization is spatially heterogeneous [9,11,17,20]; therefore, livestock treatment targets should be based on measured source-water chemistry rather than on a generalized national TDS range.

### 8.1. Effect of Water Quality on Livestock Productivity: Growth, Reproduction, and Milk Yield

Water constitutes approximately 60–70% of an adult animal’s body weight and is directly involved in digestion, thermoregulation, nutrient transport, and lactation. Dairy cows require approximately 4.5–5 kg of water per kilogram of milk produced, and inadequate water intake reduces feed consumption and milk yield [43,44,82,83,84]. Poor-quality water compounds this effect: animals offered water with elevated TDS, sulphate, or microbial contamination show further reductions in palatability and voluntary intake, amplifying productivity losses.

A study of 132 Egyptian cattle farms [24] reported associations between drinking-water quality parameters and production indicators, supporting the practical relevance of TDS, nitrate, sulphate, hardness, and microbial quality. Because this was an observational farm study, the reported correlations should not be interpreted as universal causal thresholds for Kazakhstan; they are best used to support routine water-quality monitoring and species-appropriate risk assessment.

Livestock responses to salinity depend on species, age, diet, climate, and acclimation [21,22,81]. Cattle generally tolerate lower salinity than many sheep and goat breeds, while high sulphate, nitrate, or microbiological contamination may create additional constraints even when TDS alone appears acceptable [22,23,24]. Watering-system reliability and placement also influence actual water intake and grazing distribution [85,86]. For this reason, treatment targets should be set from the complete ion profile and the intended livestock species, not from a single generic TDS cut-off.

A concise species- and parameter-specific evidence summary is provided as Supplementary Table S1. The table distinguishes guidance values from experimentally observed effects and avoids presenting uncertain numerical thresholds as universal biological limits.

### 8.2. Expansion of Utilised Pastureland Through Provision of Remote Watering Points

In arid pastoral systems, the spatial distribution of livestock grazing is tightly constrained by the location of water sources. Cattle typically do not graze more than 2–3 km from a reliable water point, while sheep and goats may extend their range to 4–6 km under favourable conditions [86]. In the vast steppe and semi-desert landscapes of Kazakhstan—where inter-source distances frequently exceed 10–20 km—large areas of potentially productive pasture remain chronically underutilised due to the absence of watering infrastructure. This represents a significant foregone productive capacity for the national livestock sector.

Installing additional watering points can redistribute grazing pressure and make previously underused areas more accessible. Rangeland studies show that livestock distribution changes measurably with distance to water, but the magnitude of pasture-utilization gain is site-specific and should not be expressed as a universal 40–60% increase [86,87,88]. For Kazakhstan, the relevant planning question is therefore how additional treated-water points alter accessible grazing area under local terrain, forage, herd, and seasonal conditions.

Modular PV-powered membrane systems have been demonstrated in remote settings and can be configured at small capacities appropriate to decentralized supply [14,15,64,70,71]. Transportability and daily production capacity should be specified for the selected commercial or pilot unit rather than assumed from a universal 10–25 m^3^/day range.

### 8.3. Reduction in Piosphere Formation and Overgrazing Around Existing Water Sources

The piosphere refers to the radial zone of vegetation degradation and soil compaction that develops around livestock watering points as a result of concentrated grazing and trampling [87]. In arid and semi-arid pastoral systems, piospheres may extend several kilometres from water sources, leading to reduced perennial vegetation cover, soil erosion, altered plant community composition, and biodiversity loss [88]. Piospheres are among the most visually prominent and ecologically damaging consequences of inadequate water infrastructure in pastoral zones.

Research in desert grassland systems has shown that the spatial distribution of grazing animals is strongly influenced by the availability and accessibility of water resources [88]. Consequently, strategically distributing multiple watering points across pastoral landscapes promotes a more even grazing pattern, reduces livestock congregation around individual water sources, and mitigates localized piosphere development, thereby facilitating vegetation recovery in previously overgrazed areas [86,87].

In the context of Kazakhstan’s steppe rangeland management, the strategic placement of new treated water sources—informed by landscape ecology, livestock movement modelling, and remote sensing-based vegetation assessment—represents a powerful tool for reversing pasture degradation while simultaneously increasing productive carrying capacity. This dual benefit positions water supply infrastructure not merely as a welfare or productivity intervention, but as a core component of sustainable land management policy.

### 8.4. Policy Implications for National Pastoral Development Programmes

The agricultural and ecological benefits documented in the preceding sections—increased livestock productivity, expanded pastureland utilization, and reduced piosphere degradation—collectively constitute a quantifiable economic case for investment in pastoral water treatment infrastructure. These benefits operate across multiple scales simultaneously: at the farm level through improved animal performance, at the landscape level through more uniform distribution of grazing pressure, and at the national level through expanded productive capacity of the pastoral sector.

Kazakhstan’s Water Code, adopted on 9 April 2025, together with broader SDG-linked land-management objectives, provides an institutional basis for linking water security with sustainable land management [2,89]. Tazhiyev et al. report that 56.5% of pastureland is underused in the context of inadequate watering infrastructure [9]. This figure quantifies the scale of the access problem, but the productive benefit of any new treatment installation should be demonstrated through site-specific monitoring of water reliability, animal performance, grazing distribution, and vegetation response rather than assumed automatically.

The linkage among water access, livestock distribution, and land condition supports treating pastoral water infrastructure as part of sustainable rangeland management rather than as a stand-alone engineering intervention [3,88,89]. Investment decisions should nevertheless distinguish documented benefits from projected national-scale outcomes and should include post-deployment monitoring of vegetation condition, grazing distribution, animal performance, and water-system reliability.

## 9. Case Studies and Regional Applications

The deployment of membrane filtration, UV disinfection, and ozonation for pastoral water supply has been documented across a range of geographical and socioeconomic contexts. Comparative analysis of these cases allows identification of transferable lessons, technology selection criteria, and implementation barriers relevant to the arid pastoral zones of Kazakhstan and Central Asia. Comparative regional case studies and their transferability to arid pastoral water supply are summarized in Table S2 (Supplementary Materials).

### 9.1. Kazakhstan: Groundwater Mineralisation, Infrastructure Gaps, and Current Initiatives

Kazakhstan presents the primary context of application for the technologies reviewed in this article. A comprehensive hydrogeological survey of the Almaty and Zhetysu regions—the first conducted in over 40 years—analysed 55 groundwater samples, reported that approximately 40% exceeded the mineralization benchmark used in that study, and noted that 56.5% of pastureland is underused in the context of limited water access [9]. Total livestock water demand in Southern Kazakhstan was estimated at 53,735 × 10^3^ m^3^/year [9]. Because WHO does not set a health-based TDS guideline value, the 40% result is treated here as a study-specific mineralization indicator rather than a universal WHO exceedance [10].

At the policy level, Kazakhstan adopted a new Water Code on 9 April 2025 (Code No. 178-VIII), establishing water security, integrated water management, conservation, and climate adaptation within the national legal framework [89]. In 2024, the government allocated KZT 218 billion (approximately USD 416 million) to 324 water-supply projects [90]. In the Mangystau Region, KazMunayGas reports that the Kenderli seawater reverse-osmosis plant, with a design capacity of 50,000 m^3^/day, was commissioned in 2025 [91]. These national-scale investments demonstrate institutional commitment to water infrastructure, while dispersed pastoral watering points still require separate evaluation of modular, decentralized treatment solutions [9,92].

An integrated assessment of Western Kazakhstan groundwater resources, combining GIS analysis with ESA Sentinel-2 remote sensing, identified that approximately 90% of assessed aquifer segments are suitable for irrigation, with saline fractions requiring NF or RO treatment [92]. This spatial differentiation—distinguishing zones where UF-only suffices from those requiring full desalination—provides an evidence base for tiered technology deployment and priority-based capital allocation, reducing unnecessary CAPEX and accelerating the expansion of treated water access across the pastoral zone.

### 9.2. Central Asia: Uzbekistan and Kyrgyzstan—Analogous Challenges and Applied Solutions

Uzbekistan faces a related but distinct water-resource challenge. CAWATER-INFO reports potential natural fresh and brackish groundwater resources of 27.6 km^3^/year, current usable groundwater of about 0.5 km^3^/year, and agricultural use of approximately 90–91% of national water resources [93]. These figures support the relevance of groundwater management and water-saving policy, but the cited source does not document a specific BWRO + UV pastoral field installation; technology-transfer claims should therefore be separated from the national water-resource statistics.

In Kyrgyzstan, World Bank rural water-supply programmememes provide an implementation and governance analogue rather than a membrane-performance case study. In January 2024, the World Bank reported that combined investments under the Sustainable Rural Water Supply and Sanitation Project and its additional financing were expected to reach 94 villages and directly benefit about 200,000 people, including 57 climate-resilient water-supply subprojects [94]. The cited program emphasizes infrastructure, storage, metering, sanitation, and institutional strengthening; it should not be described as evidence for a specific solar-BWRO + UV treatment train unless a project-level technical source documents that configuration.

Across Uzbekistan and Kyrgyzstan, the available evidence is most useful for understanding resource planning, infrastructure delivery, financing, and institutional capacity rather than for claiming a single transferable treatment technology [93,94]. These implementation lessons directly inform the recommendations of Section 10.

### 9.3. Comparative International Cases: Australia, the Middle East, and Sub-Saharan Africa

Field experience from Australia’s arid outback provides a directly relevant technical benchmark. Richards and Schäfer [70] field-tested a PV-powered hybrid UF + RO/NF system at White Cliffs, New South Wales, and produced drinking-quality water from bore water with TDS up to 3500 mg/L; reported specific energy consumption ranged from 2 to 8 kWh/m^3^ depending on feed water and operating conditions. More recent solar-RO reviews, including Almetwally et al. [71], show continuing progress in PV integration while emphasizing intermittency, storage, capital cost, and brine management as key design constraints.

The MENA region provides extensive experience with desalination under arid conditions. Reviews of conventional and renewable-energy-powered desalination document the strong influence of feed salinity, process configuration, energy source, and plant scale on energy demand and water cost [95]. At much smaller scales, SODIS can provide point-of-use microbial risk reduction in low-turbidity water [51], but it does not remove salinity and should therefore be treated as a disinfection option rather than an alternative to BWRO for mineralized groundwater.

Sub-Saharan African studies and reviews provide field evidence that SODIS can improve microbial water quality and, in some settings, reduce diarrhoeal disease burden [96,97]. These results support solar disinfection as a low-cost household or small-volume intervention, but they should not be extrapolated directly to livestock-scale water demand or to saline groundwater without additional treatment.

Collectively, the international evidence supports solar-powered BWRO with terminal UV as a strong baseline configuration when both desalination and microbial control are required, while simpler configurations may be preferable where source-water chemistry permits. International cases also show that institutional capacity, maintenance supply chains, financing, and governance are as important as nominal treatment performance for long-term success [64,70,71,93,94].

## 10. Future Research Perspectives

### 10.1. Fouling Characterisation of BWRO/NF Membranes Under Continental Arid Conditions

Published long-term fouling and scaling datasets for small BWRO/NF systems under Kazakhstan’s continental pastoral conditions are limited. High sulphate/carbonate hardness, low winter temperatures, high summer temperatures, and dust exposure can alter pretreatment and cleaning requirements, but the magnitude should be measured rather than assumed [20,39]. Multi-season pilot studies should therefore report normalized permeability, salt passage, differential pressure, scaling indices, foulant characterization, cleaning frequency, chemical consumption, and membrane replacement history.

### 10.2. Bromate Formation Potential in Central Asian Brackish Groundwaters

The preconditions for safe ozonation deployment—specifically, the characterization of source water bromide (Br^−^) at concentrations relevant to bromate (BrO_3_^−^) formation—remain unmet across the majority of Kazakhstan’s pastoral aquifer zones. The relationship between ozone dose, pH, temperature, dissolved organic carbon, and bromate yield under the hydrochemical conditions specific to pre-Caspian, Aral Sea basin, and southern steppe groundwaters has not been empirically established. A targeted field sampling program (minimum 150 sites, encompassing seasonal variation) combined with controlled laboratory ozonation trials using authentic source waters would generate the bromate formation potential models required for regulator-acceptable risk assessment and the safe application of O_3_/UV advanced oxidation processes [54,68]. This gap is particularly urgent given the growing interest in ozonation as a tool for addressing agricultural micropollutant contamination in Central Asian groundwater.

### 10.3. Long-Term Field Performance of Solar-BWRO in Continental Climates

The technology transfer case for solar-powered BWRO in Kazakhstan rests primarily on performance data from coastal arid zone installations in Australia, the MENA region, and sub-Saharan Africa. Continental arid climates impose qualitatively different operational stresses: photovoltaic output is reduced 10–40% by dust deposition without regular panel cleaning; membrane integrity may be compromised by freeze–thaw cycling during winter shutdowns; and inter-site logistics for replacement membranes and chemical consumables are substantially more constrained than in coastal deployment contexts [36,71]. Multi-year monitored field trials at representative Kazakhstani pastoral sites—reporting actual energy production, membrane degradation rates, system availability, O&M labour requirements, and total lifecycle costs under operational rather than laboratory conditions—represent the single most impactful research investment for de-risking scaled national deployment and attracting private sector participation.

### 10.4. Antimicrobial Resistance Genes as Emerging Contaminants in Pastoral Water Sources

UV irradiation and ozonation have demonstrated the capacity to reduce antimicrobial resistance genes (ARGs) in water treatment systems; however, their performance and dose–response relationships in brackish groundwater matrices remain poorly understood because most available studies have been conducted using municipal wastewater and drinking water systems [98]. Defining treatment efficacy benchmarks for ARG removal is a prerequisite for incorporating antimicrobial resistance control into regulatory frameworks for pastoral water supply.

### 10.5. Performance of O_3_/Membrane Hybrid Systems Under Variable Pastoral Source Water Quality

Laboratory and pilot literature indicates that ozone pretreatment can modify organic matter and sometimes improve downstream membrane performance, but reported benefits are matrix- and process-dependent [42,43,68]. Whether that benefit outweighs bromate risk, added energy, and maintenance under Central Asian pastoral conditions remains unresolved. Comparative pilots should therefore test O_3_/UF or O_3_/BWRO against simpler coagulation/filtration pretreatment using the same feed water and common performance metrics.

### 10.6. Socioeconomic Determinants of Long-Term Technology Adoption and Sustainability

The international case literature reviewed in Section 9 indicates that institutional capacity, O&M supply chains, operator retention, financing, and governance can determine the sustainability of decentralized rural water systems [64,94]. Research questions that remain unanswered in the Central Asian pastoral context include which cost-recovery mechanisms are viable at watering-point scale, which governance models best sustain small BWRO systems over their lifecycle, and which operator-training arrangements produce durable local capacity. Interdisciplinary research integrating water engineering, environmental economics, and social science is therefore required.

### 10.7. AI-Assisted and Remote Sensing-Integrated Monitoring for Pastoral Water Management

The integration of satellite remote sensing products, including groundwater salinity proxies derived from Sentinel-2 imagery, vegetation indicators, and field hydrochemical data, can support prioritization and monitoring of pastoral water resources in Kazakhstan [92]. Coupling those data with telemetry from decentralized treatment systems may enable predictive maintenance and optimization of water storage and solar-energy use. Such digital monitoring is consistent with Kazakhstan’s broader water-sector modernization and digitalization initiatives, but it requires validation under the communications and maintenance constraints of remote pastoral areas [90].

### 10.8. Emerging and Alternative Treatment Technologies

Beyond MF, UF, NF, BWRO, UV, and ozonation, several electro-membrane and thermally driven processes may be relevant to selected brackish-water sources. Their suitability should be judged by salinity, target ion removal, energy source, recovery, concentrate management, and field maturity rather than by novelty alone [14,15,99,100].

Electrodialysis (ED) is an established electro-membrane desalination process for brackish water. Electrodialysis reversal (EDR) is a commercial operating mode of ED in which polarity is periodically reversed to reduce scaling and fouling; it is therefore more precise to discuss ‘ED, including EDR operation’ rather than treat EDR as a separate desalination principle [14,15]. ED/EDR can be attractive at lower brackish salinities where partial demineralisation is sufficient. Membrane distillation (MD) can couple desalination with low-grade or solar thermal heat but remains energy- and module-design sensitive. Membrane capacitive deionisation (MCDI/CDI) is most competitive at relatively low salinity, while forward osmosis (FO) still requires regeneration of the draw solution and is therefore not intrinsically low-energy [100].

ED/EDR is commercially mature for appropriate brackish-water applications, but field evidence for small solar-powered pastoral installations in Kazakhstan is currently limited. MD, CDI/MCDI, and FO warrant targeted pilot evaluation only where their process advantages match the source-water problem. Future comparisons should report specific energy consumption, recovery, salt rejection or selectivity, fouling, concentrate volume, maintenance, and lifecycle cost against a BWRO baseline.

## 11. Conclusions

This review examined the scientific basis, operational characteristics, and practical applicability of membrane filtration, ultraviolet disinfection, ozonation, and integrated treatment trains for pastoral water supply in arid and semi-arid regions, with particular emphasis on Kazakhstan and Central Asia. The analysis demonstrates that technology selection should be based primarily on source-water chemistry, required treatment capacity, energy availability, operational constraints, and the intended use of the treated water.

For groundwater requiring substantial desalination, brackish water reverse osmosis represents the most robust baseline option because of its high rejection of dissolved salts and its compatibility with photovoltaic power supply. Nanofiltration may provide a more energy-efficient alternative where selective removal of hardness, sulphate, and multivalent ions is sufficient. Microfiltration and ultrafiltration are most appropriate as pretreatment and microbial-barrier processes rather than as desalination technologies. Low-pressure ultraviolet irradiation is particularly suitable as a final disinfection step because it requires minimal chemical handling and can be readily integrated into decentralized systems. Ozonation has greater value where oxidation of iron, manganese, natural organic matter, or specific micropollutants is required, although its application demands more complex operation and careful control of bromate formation.

The review also shows that water-treatment performance cannot be evaluated independently of pastoral management objectives. Improved water quality can support livestock health and productivity, while appropriately distributed watering points can improve access to grazing resources and reduce excessive concentration of animals around a limited number of water sources. Nevertheless, the magnitude of these benefits is strongly site-dependent and should not be generalized across different livestock species, hydrogeological settings, climates, or grazing systems. Consequently, productivity gains, pasture expansion, and piosphere recovery should be quantified through local field monitoring rather than assumed from results obtained under different conditions.

The long-term success of decentralized pastoral water-treatment systems depends not only on treatment efficiency but also on maintenance capacity, availability of spare parts and consumables, operator training, financing mechanisms, and institutional responsibility. These factors should therefore be incorporated into technology selection and project appraisal from the initial design stage. Solar-powered treatment systems are especially promising for remote locations, but their sizing must account for seasonal irradiation, dust deposition, storage requirements, treatment demand, and the energy intensity of the selected process.

Overall, decentralized water treatment for pastoral areas represents an important intersection of water security, livestock production, renewable energy use, and sustainable land management. For Kazakhstan, large-scale deployment should proceed through monitored field demonstrations representing different hydrochemical and climatic zones, followed by standardized technical and economic evaluation. Rather than promoting a single universal treatment configuration, future implementation should rely on adaptable treatment trains selected according to local groundwater quality, livestock requirements, energy resources, logistics, and environmental constraints. Such an evidence-based approach would provide a more reliable foundation for the sustainable expansion of pastoral water infrastructure across Kazakhstan and other arid regions of Central Asia.

## Figures and Tables

**Figure 1:**
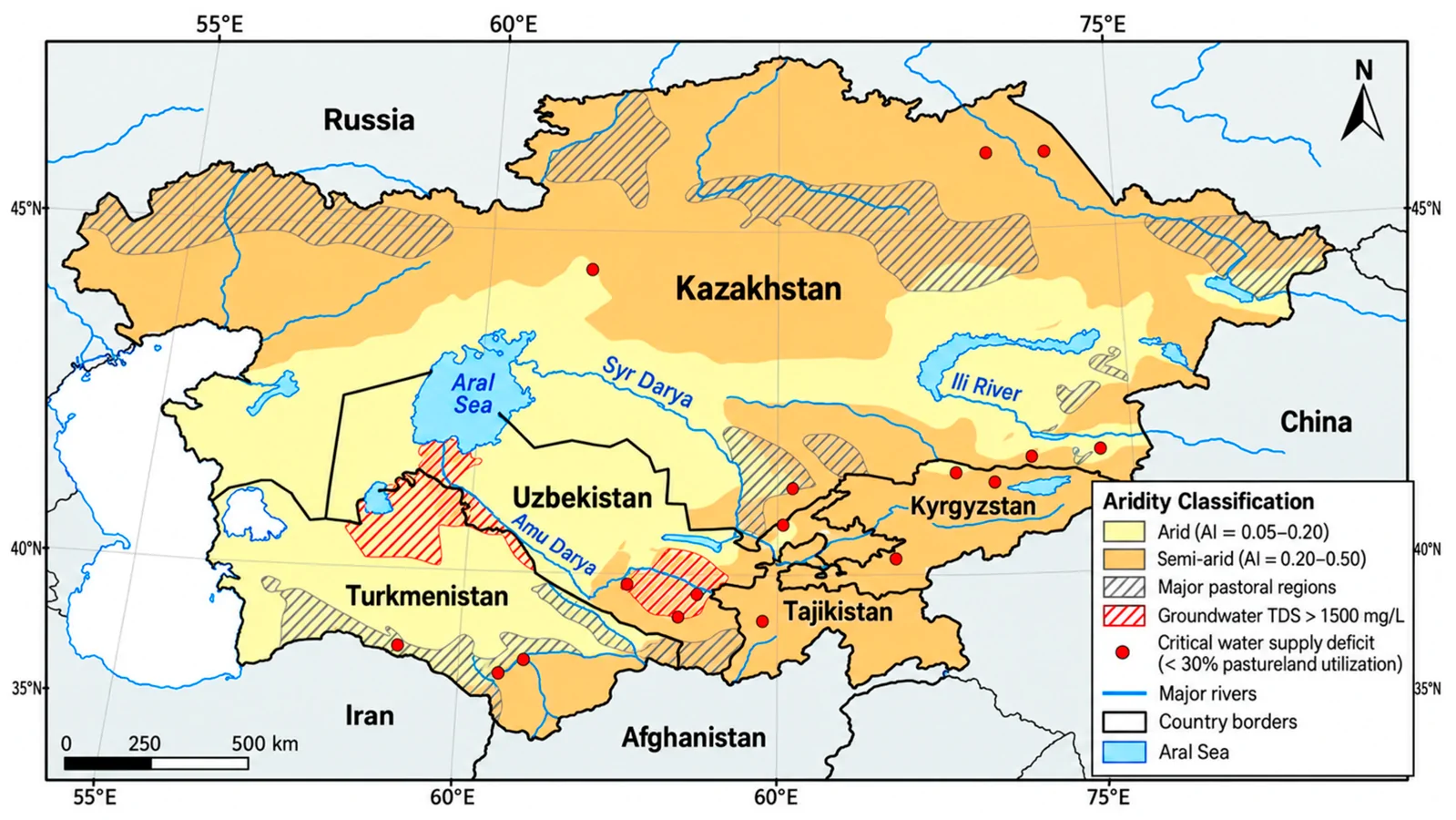
Map of arid and semi-arid pastoral zones in Central Asia. Adapted from Tazhiyev, S. et al. [9], Water 2025, 17, 1297 (https://doi.org/10.3390/w17091297), licensed under CC BY 4.0 The Aridity Index (AI) is defined as the ratio of mean annual precipitation (P, mm) to mean annual potential evapotranspiration (PET, mm): AI = P/PET, following the UNEP Global Aridity Index framework [1]. Values below 0.05 designate hyperarid zones, 0.05–0.20 arid zones, and 0.20–0.50 semi-arid zones; the pastoral territories of southern and western Kazakhstan fall predominantly within the arid and semi-arid categories (AI = 0.05–0.45), consistent with the hydrological deficit depicted in Figure 1 and the treatment challenge addressed in this review.

**Figure 2:**
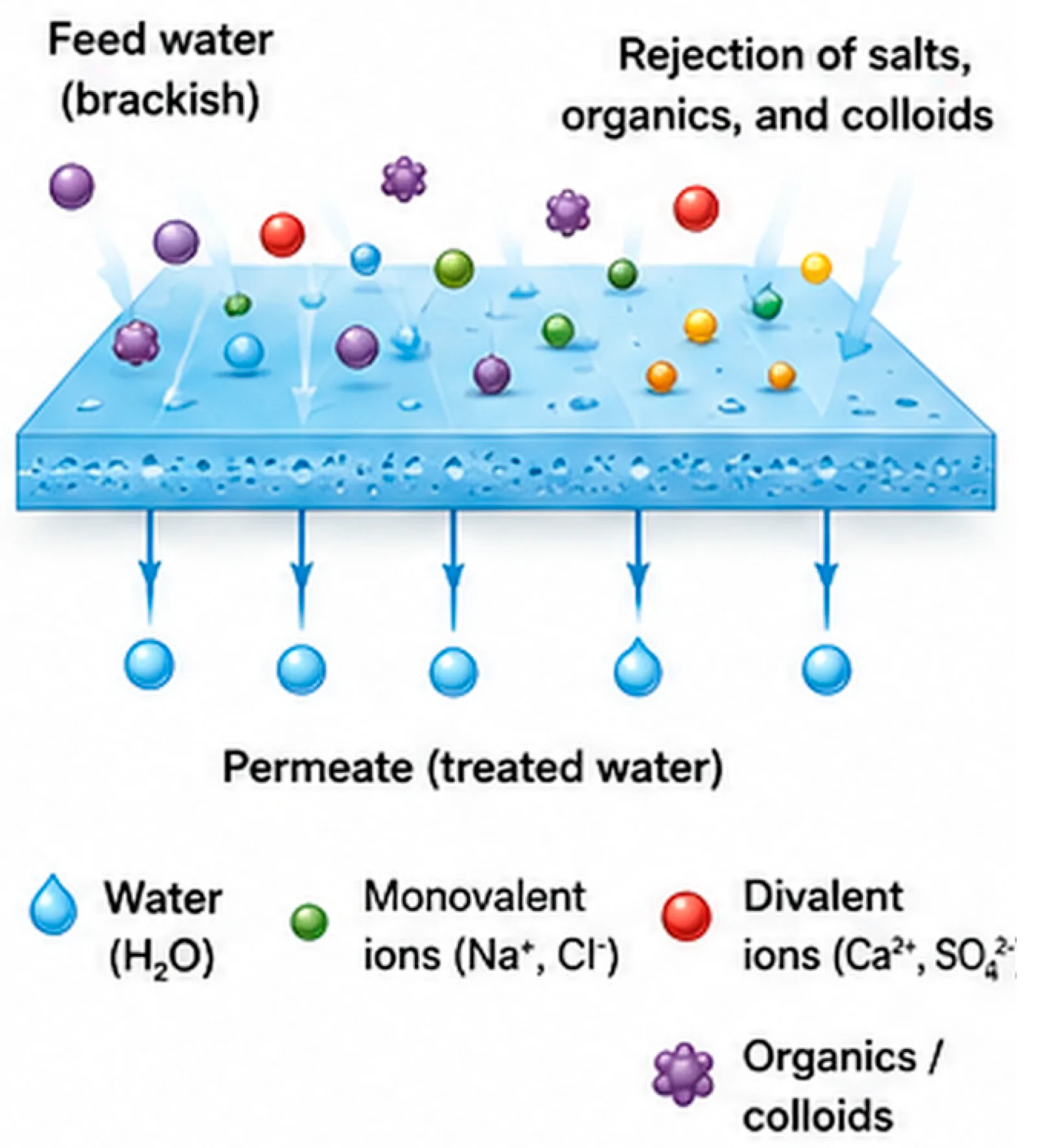
Schematic of the NF/RO membrane separation mechanism, showing rejection of salts, organics, and colloids from brackish feed water and production of a treated permeate stream.

**Figure 3:**
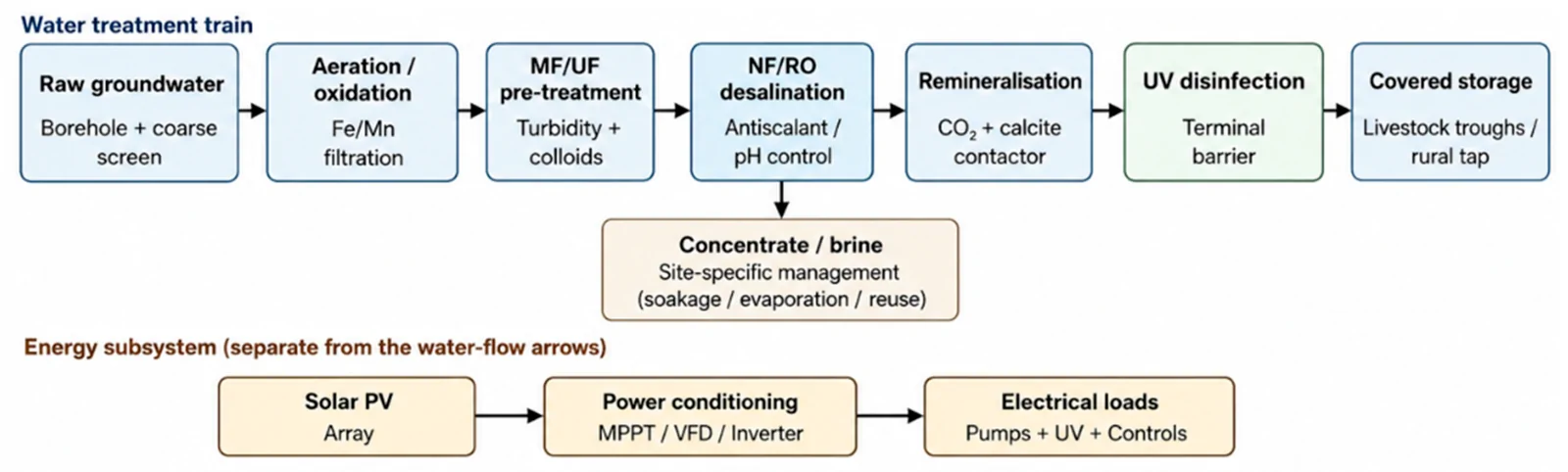
Simplified treatment train for a solar-powered pastoral groundwater system. Water-flow arrows connect only consecutive treatment stages. The concentrate stream branches from NF/RO to site-specific management, while the PV subsystem is shown separately as the source of electrical power for pumps, UV, and controls.

**Figure 4:**
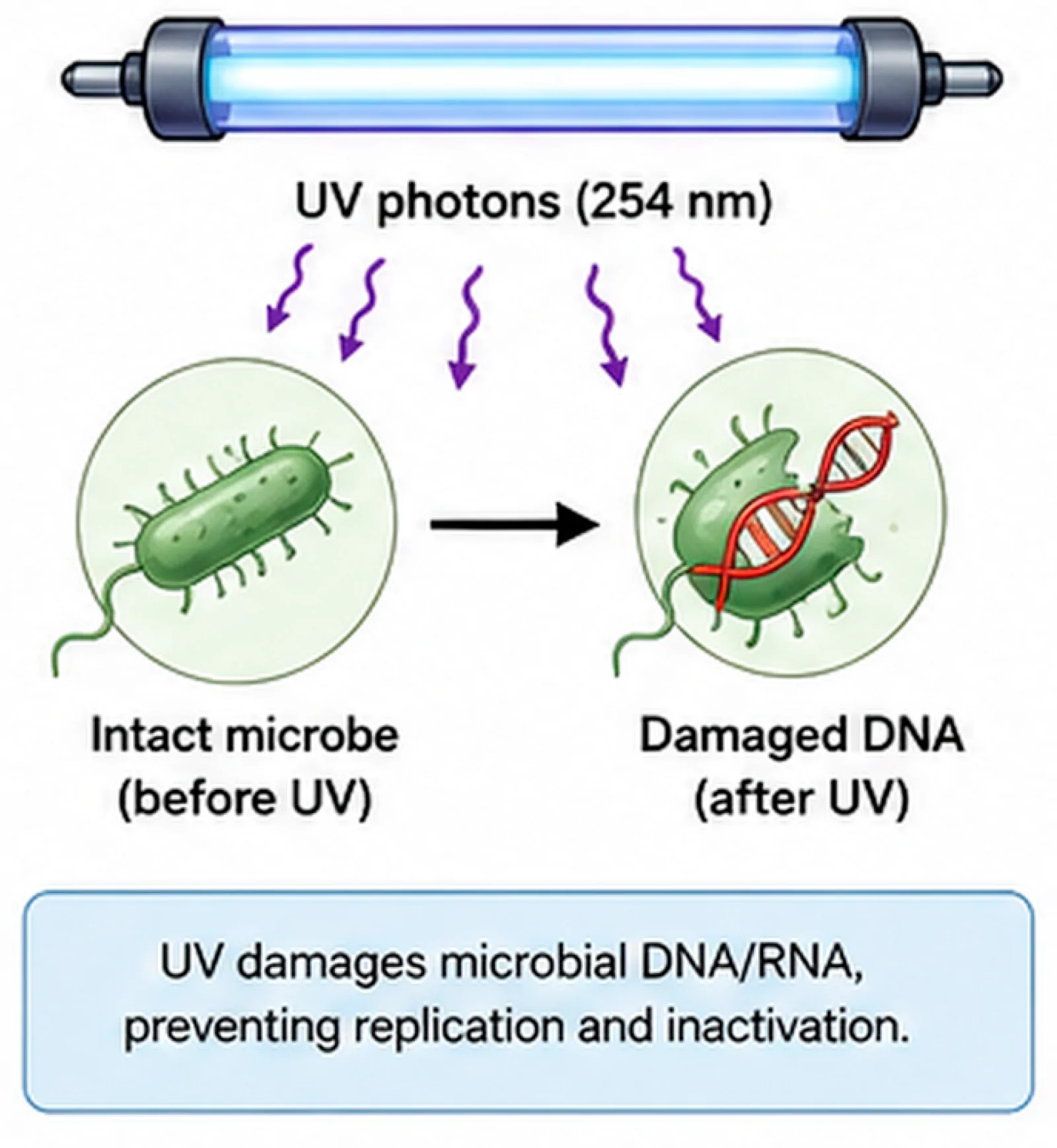
UV disinfection mechanism: UV-C photons (254 nm) penetrate the microbial cell and damage nucleic acid (DNA/RNA), preventing replication and inactivating the pathogen.

**Figure 5:**
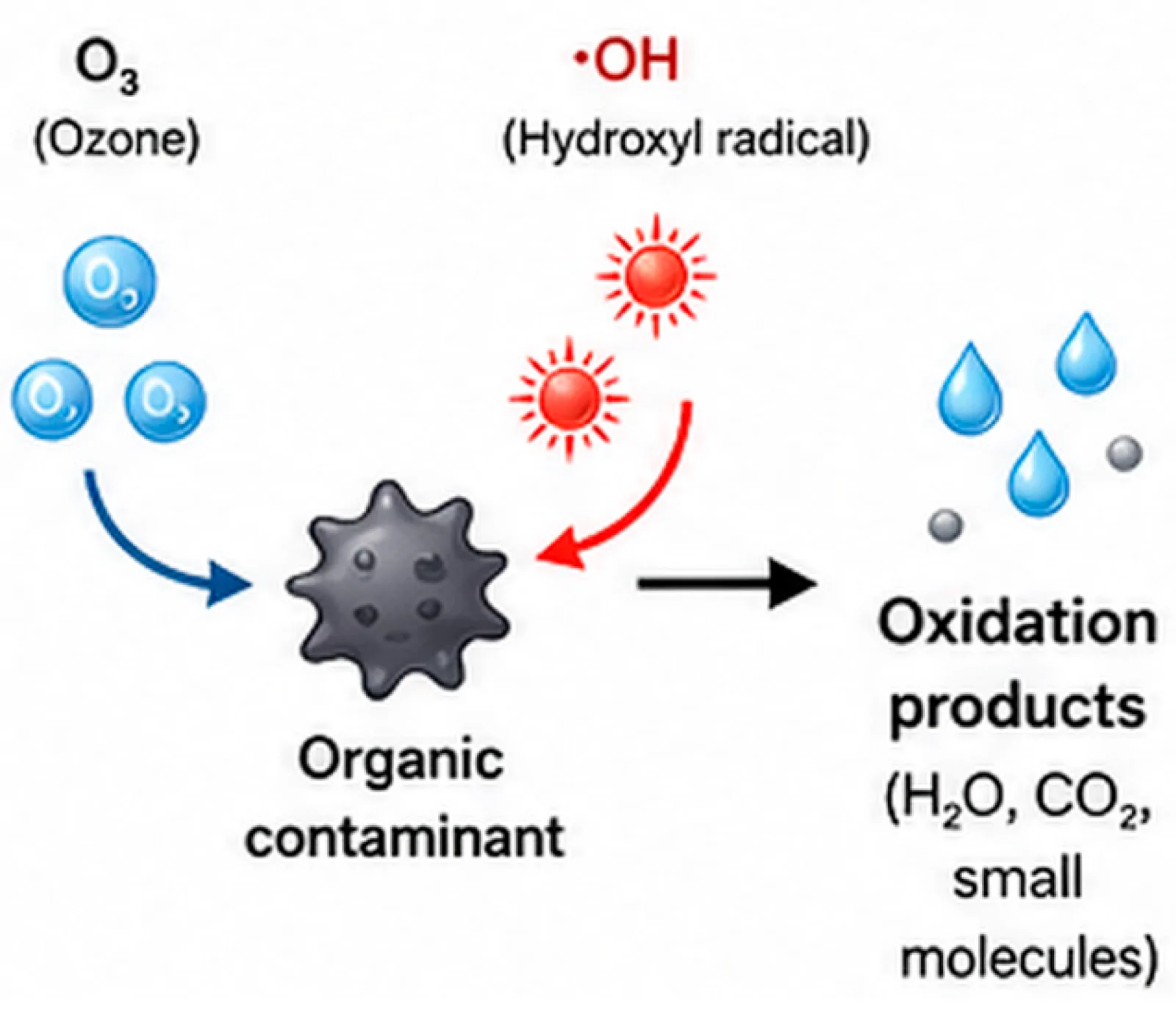
Ozonation mechanism: molecular ozone (O_3_) and secondary hydroxyl radicals (•OH) oxidize organic contaminants, yielding oxidation products such as H_2_O and CO_2_.

**Figure 6:**
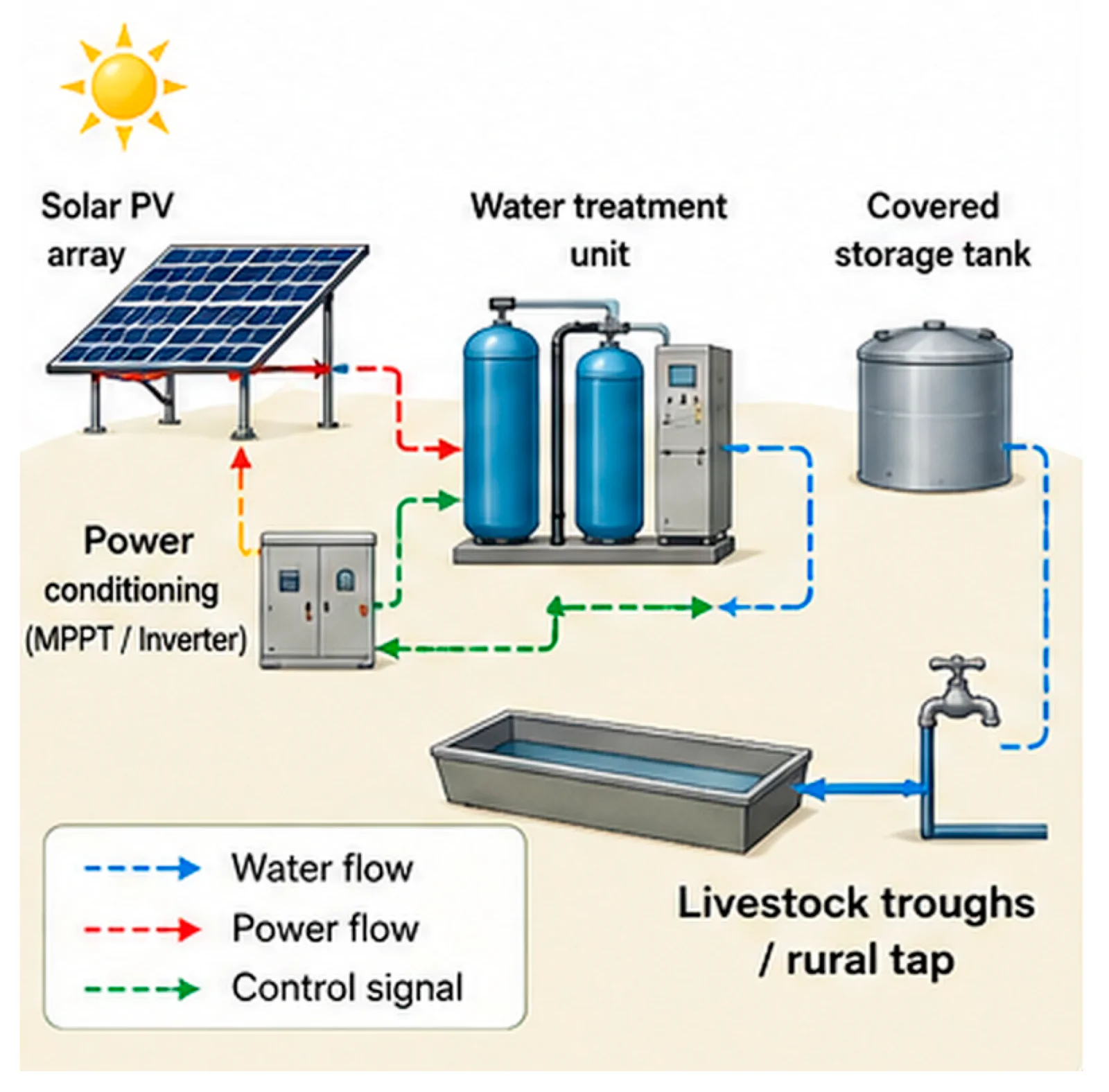
Conceptual layout of an off-grid solar-powered pastoral water system, showing the solar PV array, power conditioning, water treatment unit, covered storage tank, and livestock trough, with water-flow, power-flow, and control-signal paths indicated.

**Table 1:** Selected water-quality guidance and national limits relevant to rural drinking-water supply and livestock watering: WHO (2026), Kazakhstan SanPiN (2023), and livestock-watering guidance.

Parameter	WHO (2026) Drinking Water	Kazakhstan SanPiN (2023)	FAO Livestock (Cattle/Sheep)	Remarks
TDS (mg/L)	No health-based GV; palatability generally good below ~600 mg/L and increasingly impaired above ~1000 mg/L	≤1000	≤3000–7000	TDS is an acceptability/mineralization indicator; individual constituents determine health risk
pH	No health-based GV; important operational parameter	6.0–9.0	6.0–9.0	pH affects treatment, corrosion, disinfection chemistry, and acceptability
Nitrate NO_3_^−^ (mg/L)	≤50 mg/L as NO_3_^−^	≤45	≤100–400	Health-based guideline value; infant methaemoglobinaemia is the critical endpoint
Sulphate SO_4_^2−^ (mg/L)	No health-based GV; acceptability concern at high concentrations	≤500	≤1000	High sulphate can impair acceptability and may have laxative effects
Chloride Cl^−^ (mg/L)	No health-based GV; taste may be impaired at ~250 mg/L	≤350	≤1000	Primarily an acceptability/corrosion consideration at typical drinking-water concentrations
Fluoride F^−^ (mg/L)	≤1.5 mg/L	≤1.5	≤2.0	Health-based guideline value; fluorosis risk increases with long-term excess exposure
Iron Fe (mg/L)	No health-based GV; acceptability/staining concern	≤0.3	≤0.3–1.0	Iron can cause colour, staining and treatment/fouling problems
Turbidity (NTU)	No health-based GV; large supplies should achieve ≤ 0.5 NTU before disinfection as an operational target	≤2.6	≤5	Turbidity interferes with treatment and disinfection; 4 NTU is a visibility/acceptability level, not a WHO health limit
Total coliforms (CFU/100 mL)	Operational indicator; not the primary faecal indicator	0	0	Use mainly for operational/distribution-system assessment rather than as a universal health-based zero limit
*E. coli* (CFU/100 mL)	Not detectable in any 100 mL sample	0	0	Primary indicator of faecal contamination

Sources: WHO Guidelines for Drinking-water Quality, 4th ed. incorporating the 1st, 2nd and 3rd addenda, 2026 [10]; Kazakhstan SanPiN Order No. 26, 2023 [27]; FAO Water Quality for Agriculture [22]; Australian and New Zealand Livestock Drinking Water Guidelines [23]. WHO entries distinguish health-based guideline values from acceptability or operational guidance.

**Table 2:** Selected source-specific groundwater-quality findings relevant to pastoral water-treatment decisions in Kazakhstan.

Region/Study Area	Study Basis	Mineralization/TDS Finding	Other Reported Findings	Relevance to Treatment Selection	Source
Almaty & Zhetysu, Southern Kazakhstan	55 groundwater samples plus field/hydrogeological assessment	≈40% of samples exceeded the mineralization benchmark used in the study	56.5% of pastureland underused in the context of limited water access; livestock water demand quantified	A substantial subset of sources requires source-specific assessment; do not treat the study benchmark as a WHO health-based TDS limit	[9]
Zhambyl Region, Southern Kazakhstan	204 wells investigated; field survey plus hydrogeological and laboratory assessment	Mineralization and groundwater quality vary by district and individual well; no single regional concentration range is reported here	Study identifies groundwater depletion, pollution, waterlogging and salinization as regional concerns	Use well-specific chemistry and health-risk parameters rather than synthetic regional averages	[11]
Balkhash District, Almaty Region	Centralized supplies and tube wells in nine villages	Centralized water met Kazakhstan mineralization limits; selected well waters exceeded them, including > 6× the standard in Akkol	Selected wells also showed elevated boron, iron and/or uranium; six of nine villages met standards for both source types	Decentralized wells can require site-specific treatment even where centralized supply complies	[28]

Note: Table 2 reports only findings that are directly traceable to the cited study. It intentionally avoids converting heterogeneous study designs into artificial regional ranges. WHO does not establish a health-based guideline value for TDS [10].

**Table 4:** UV fluence requirements (mJ/cm^2^) for inactivation of key waterborne pathogens at 254 nm: summary of dose–response data from collimated beam studies.

Microorganism	1 log (mJ/cm^2^)	2 log (mJ/cm^2^)	3 log (mJ/cm^2^)	4 log (mJ/cm^2^)	Remarks
*Escherichia coli*	1–2	3–5	6–10	10–16	Most sensitive indicator; used for biodosimetry calibration
Total coliforms	2–4	5–8	10–15	16–22	Slightly more resistant than *E. coli*; standard surrogate in practice
*Salmonella typhi*	2–3	5–8	10–14	15–20	Enteric pathogen; inactivated at doses < 20 mJ/cm^2^
*Cryptosporidium parvum* (oocysts)	<1	<3	<10	<22	Highly sensitive to UV; completely resistant to chlorination at normal doses
*Giardia lamblia* (cysts)	<1	<5	<10	<11	>4 log achieved at dose < 10 mJ/cm^2^; does not repair UV damage
MS2 coliphage (virus surrogate)	14–18	30–40	50–60	80–100	Most UV-resistant common surrogate; conservative design basis for viruses
Adenovirus (human)	20–30	40–60	80–120	>150	Most UV-resistant human enteric virus; governs design for virus credit

Sources: Hijnen et al. [13]; Linden et al. [47]; WHO Guidelines for Drinking-water Quality [10]; Blatchley & Peel [45]. Values represent approximate median estimates from collimated beam studies; actual reactor doses must account for hydraulic efficiency (RED/RD ratios from biodosimetry).

**Table 5:** Decision matrix for terminal disinfection technology selection in pastoral water supply systems.

Parameter	UV Disinfection (LP)	Ozonation	Chlorination
Residual disinfection capacity	None	None (decays rapidly)	Yes—controls regrowth in storage and distribution
Chemical storage/handling	None required	On-site O_3_ generation; no storage	Cl_2_ gas or NaOCl—requires safe storage and handling
pH dependence of efficacy	None—effective pH 4–10	Slight—OH yield increases with pH	Strong—HOCl/OCl^−^ equilibrium (pKa 7.5); less effective at pH > 7.5
Turbidity/UVT sensitivity	High—UVT_254_ ≥ 75% required; pre-treatment essential	Low—not UVT-dependent	Low—not UVT-dependent
DBP formation potential	None (no organic DBPs)	Bromate if Br^−^ > 0.1 mg/L; aldehydes from NOM oxidation	THMs and HAAs from NOM reaction; regulated carcinogens
Cryptosporidium/Giardia efficacy	Excellent—>3 log at <10 mJ/cm^2^	Giardia: EPA 3-log ozone CT = 0.72 mg·min/L at 20 °C, pH 6–9; Cryptosporidium: EPA 3-log CT = 12 mg·min/L at 20 °C and increases at lower temperature [52]	Ineffective at practicable doses
Solar-powered off-grid suitability	Excellent—0.02–0.05 kWh/m^3^; simple PV integration	Moderate—0.10–0.30 kWh/m^3^; ancillary equipment required	Suitable only as supplementary residual agent post-UV or post-O_3_

Note: UV disinfection is the recommended primary disinfection technology for remote, off-grid pastoral installations. Chlorination is recommended as a supplementary residual disinfectant for post-treatment storage where water cannot be used immediately. Ozonation is indicated selectively for larger-capacity systems where its oxidative capabilities for Fe/Mn removal and micropollutant control provide additional benefits. Sources: WHO [10]; von Gunten [53]; Richardson et al. [50]; Oguma et al. [48].

**Table 6:** Second-order rate constants for reactions of O_3_ and •OH with key contaminants, and CT values for pathogen inactivation relevant to pastoral water treatment system design.

Target/Reaction	k(O_3_) (M^−1^s^−1^)	k(•OH) (M^−1^s^−1^)	Relevance to Pastoral Water Treatment
Fe^2+^ oxidation by O_3_	8.2 × 10^5^	3.5 × 10^8^	Rapid Fe^2+^ oxidation; hydrolysis/precipitation forms filterable Fe(OH)_3_
Mn^2+^ oxidation by O_3_	1.5 × 10^3^	2.6 × 10^7^	Oxidation can form particulate MnO_2_ requiring downstream filtration
Humic substances/NOM	10^2^–10^4^	~10^8^	Oxidation changes NOM character; effect is matrix-dependent
Bromide oxidation pathway	160	1.1 × 10^9^	Can lead to bromate; source Br^−^ and operating conditions must be characterized
*E. coli* inactivation (CT concept)	very low CT	n/a	Ozone is highly effective against vegetative bacteria; CT depends on conditions
Giardia cyst inactivation	CT = 0.72 mg·min/L	n/a	EPA 3-log Giardia CT at 20 °C, pH 6–9; 1.90 mg·min/L at 5 °C [52]
Cryptosporidium oocyst inactivation	CT = 12 mg·min/L at 20 °C	n/a	EPA LT2 3-log Cryptosporidium CT; 19 mg·min/L at 15 °C and 47 mg·min/L at 5 °C. Report pH and source-water conditions [52]
Micropollutant degradation	10^1^–10^6^ (variable)	10^8^–10^10^	Relevant only when source characterization identifies susceptible trace organics

Sources: [52,53,54,55,56,57,58,59,60,61]. Reaction-rate constants refer to the conditions reported in the cited literature. For Giardia, the EPA ozone CT values shown here apply at pH 6–9 and are temperature-dependent [52]. For Cryptosporidium, the EPA LT2 3-log CT is 12 mg·min/L at 20 °C, 19 mg·min/L at 15 °C, and 47 mg·min/L at 5 °C [52]; pH and source-water conditions should still be reported because they affect ozone stability and by-product formation.

**Table 7:** Comparative techno-economic parameters of water treatment technologies for pastoral water supply in arid zones.

Technology	CAPEX (USD/m^3^·d^−1^)	OPEX (USD/m^3^)	Energy (kWh/m^3^)	Membrane/Lamp Life	Principal Application in Pastoral Context
UV disinfection (LP)	150–400	0.05–0.15	0.02–0.08	UV lamp: 1–2 yr	Terminal disinfection, post-RO polishing
Ozonation	300–800	0.20–0.50	dose- and system-dependent; generator load typically lower than BWRO	Ozone gen: 10–15 yr	Organic micropollutant & colour removal
UF (ultrafiltration)	400–900	0.25–0.55	0.05–0.15	3–7 yr	Turbidity removal, pre-RO; pathogen barrier
NF (nanofiltration)	600–1200	0.35–0.80	0.20–0.60	3–5 yr	Hardness/sulphate reduction
BWRO (brackish RO)	700–1500	0.40–1.20	0.80–2.50	3–7 yr	TDS and mineralization reduction; main desalination step
Solar-PV + BWRO + UV	900–2000	0.30–1.50	≈1–3 (system electricity; supplied by PV)	PV: 25 yr; RO: 5 yr	Off-grid pastoral watering points; SDG 6/7 nexus
O_3_/UV AOP	800–1500	0.50–1.20	0.30–0.70	—	Refractory organics, disinfection by-product control

## Data Availability

No new data were created or analysed in this review. Data and quantitative values discussed in the manuscript are available from the cited sources.

## Supplementary Materials

The following supporting information can be downloaded at: https://www.mdpi.com/article/10.3390/membranes16100331/s1. Table S1: Species- and parameter-specific evidence relevant to livestock water-quality targets, water intake, and productivity outcomes. Table S2: Comparative case studies of water treatment technology deployment in arid and semi-arid pastoral and rural contexts. References [9,21,22,23,35,44,45,52,71,72,82,83,84,85,86,87,88,89,91,92,93,94,95,96,97,98] are cited in the Supplementary Materials.

## Abbreviations

The following abbreviations are used in this manuscript:

Abbreviation	Full Name
AI	Aridity Index
AOP	Advanced Oxidation Process
ARG	Antimicrobial Resistance Gene
BAC	Biological Activated Carbon
BDOC	Biodegradable Dissolved Organic Carbon
BWRO	Brackish Water Reverse Osmosis
CAPEX	Capital Expenditure
CDI	Capacitive Deionisation
CIP	Cleaning-In-Place
CPD	Cyclobutane Pyrimidine Dimer
CT	Concentration × Time (disinfection credit)
DBP	Disinfection By-Product
DOC	Dissolved Organic Carbon
DSPM-DE	Donnan–Steric–Pore and Dielectric Exclusion model
ED/EDR	Electrodialysis/Electrodialysis Reversal
FAO	Food and Agriculture Organization
FO	Forward Osmosis
GWP	Global Warming Potential
HAA	Haloacetic Acid
HDPE	High-Density Polyethylene
HWTS	Household Water Treatment and Safe Storage
IARC	International Agency for Research on Cancer
LCA	Life Cycle Assessment
LP	Low-Pressure (UV lamp)
MD	Membrane Distillation
MF	Microfiltration
MP	Medium-Pressure (UV lamp)
MWCO	Molecular Weight Cut-Off
NF	Nanofiltration
NOM	Natural Organic Matter
O_3_	Ozone
OPEX	Operational Expenditure
PET	Polyethylene Terephthalate
PS-SODIS	Photosensitiser-Enhanced Solar Disinfection
PV	Photovoltaic
RO	Reverse Osmosis
ROS	Reactive Oxygen Species
SanPiN	Sanitary and Epidemiological Norms and Rules (Kazakhstan)
SDG	Sustainable Development Goal
SODIS	Solar Water Disinfection
TDS	Total Dissolved Solids
TFC	Thin-Film Composite
THM	Trihalomethane
TMP	Transmembrane Pressure
UF	Ultrafiltration
UNCCD	United Nations Convention to Combat Desertification
UV	Ultraviolet
UV-LED	Ultraviolet Light-Emitting Diode
UVT	Ultraviolet Transmittance
VFD	Variable Frequency Drive
WHO	World Health Organization
ZLD	Zero Liquid Discharge
OH	Hydroxyl Radical
