# Supplementary material for: Membrane-Based Solar-Powered Brackish Water Treatment for Pastoral Livestock Watering in Arid Kazakhstan: A Techno-Economic Decision Framework
Source: Membranes (Basel). 2026 Oct 2;16(10):331. doi: 10.3390/membranes16100331 (PMC13707216; doi:10.3390/membranes16100331)
Supplement: Supplementary file 1 [file membranes-16-00331-s001.zip › membranes-4469659-supplementary.pdf]

membranes

MDPI

*Review*

# Membrane-Based Solar-Powered Brackish Water Treatment for Pastoral Livestock Watering in Arid Kazakhstan: A Techno-Economic Decision Framework

**Timur Sh. Ustabaev [1], Gulzhan S. Alimbetova [1,*], Bakhtiyar Zh. Ismailov [1], Berik M. Zhaksybek [1], Ivan V. Basmanov [1], Anar Z. Batynova [2] and Marzhan S. Kalmakhanova [2,*]**

[1] Kazakh Scientific Research Institute of Water Management LLP, Taraz 00008, Kazakhstan; timoha_85_85@mail.ru (T.S.U.); bakhtiyarismailov2016@mail.ru (B.Z.I.); beka.1999.w@gmail.com (B.M.Z.); ivan@mail.ru (I.V.B.)
[2] Department of Chemistry and Chemical Technology, M. Kh. Dulaty Taraz University, Taraz 080012, Kazakhstan; g.a.kabdulova@gmail.com
* Correspondence: gulzhan-alimbetova@mail.ru (G.S.A.); marjanseitovna25@gmail.com (M.S.K.)

**Table S1. Species- and parameter-specific evidence relevant to livestock water-quality targets, water intake, and productivity outcomes.**

| Evidence type | Species / context | Parameter or condition | Evidence or guidance summarized in the review | Interpretation for pastoral water treatment | Source(s)* |
|---|---|---|---|---|---|
| Guidance | Cattle and sheep; livestock watering | Total dissolved solids (TDS) / salinity | International livestock-watering guidance allows substantially higher salinity than human drinking-water guidance; the review summarizes an indicative livestock range of approximately 3,000–7,000 mg/L, depending on species and conditions. | Use as a screening range only. Final targets should account for species, age, diet, climate, acclimation, and the full ion profile rather than TDS alone. | [21,22] |
| Guidance | Livestock watering | Nitrate, sulphate and other ions | The review summarizes livestock-oriented guidance of approximately 100–400 mg/L nitrate (as $NO_3^-$) and up to about 1,000 mg/L sulphate; other constituents such as chloride, fluoride and iron also require parameter-specific assessment. | Do not infer safety from TDS alone. A source with moderate TDS may still require treatment if nitrate, sulphate, fluoride, iron or other constituents are elevated. | [21,22] |
| Experimental study | Growing and mature Blackhead Ogaden sheep and Somali goats | Increasing drinking-water salinity | Controlled experimental evidence shows that salinity level can affect water intake and loss, feed utilization, body weight, thermoregulatory traits and blood constituents. Responses vary with species and physiolog- | Supports species-specific treatment targets and cautions against transferring one salinity threshold across all livestock classes. | [82] |

| Evidence type | Species / context | Parameter or condition | Evidence or guidance summarized in the review | Interpretation for pastoral water treatment | Source(s)* |
|---|---|---|---|---|---|
| | | | ical stage. | | |
| Observational farm evidence | Cattle; 132 farms | TDS, nitrate, sulphate, hardness and microbial water quality | Associations were reported between drinking-water quality parameters and cattle-farm production indicators. | Useful for routine monitoring and risk prioritization, but observational associations should not be presented as universal causal thresholds for Kazakhstan. | [23] |
| Nutritional / extension guidance | Dairy cattle | Water quantity and milk production | The review notes a requirement of approximately 4.5–5 kg of water per kilogram of milk produced; inadequate water intake can reduce feed intake and milk yield. | Treatment capacity must satisfy both water-quality targets and peak daily water demand. Improving quality without providing adequate quantity will not deliver the expected productivity benefit. | [44,45,83–85] |
| Guidance / practical evidence | Beef and dairy cattle | Palatability and voluntary intake | Poor salinity, sulphate, nitrate or microbial quality can reduce palatability and voluntary intake, compounding productivity losses. | Water-quality assessment should integrate chemical and microbiological parameters with animal intake behavior and seasonal demand. | [21–23,44,45] |
| Grazing-management guidance | Cattle versus sheep/goats | Distance to reliable watering point | The review cites typical grazing distances of approximately 2–3 km for cattle and 4–6 km for sheep and goats under favorable conditions. | Water-point spacing affects realized intake, grazing distribution and access to forage; treatment-system siting should therefore be integrated with rangeland planning. | [86] |
| Field / rangeland evidence | Grazing livestock and rangeland systems | Water-point placement and grazing distribution | Field evidence shows that placement and availability of water sources measurably affect livestock distribution and the spatial concentration of grazing pressure. | Do not assume a universal percentage increase in pasture utilization. Benefits should be quantified through site-specific monitoring after new watering points are installed. | [87–89] |

** Reference numbers correspond to the current manuscript numbering.*

**Important interpretation note.** Guidance values and observed experimental/field effects are intentionally separated. The table should not be used to present uncertain numerical thresholds as universal biological limits across species, climates, diets, or acclimation conditions.

## Supplementary Materials

### Table S2. Comparative case studies of water treatment technology deployment in arid and semi-arid pastoral and rural contexts.

| Region / Country | Problem context | Technology applied | Key findings and transferability to arid pastoral water supply | Key sources |
|---|---|---|---|---|

| Region / Country | Problem context | Technology applied | Key findings and transferability to arid pastoral water supply | Key sources |
|---|---|---|---|---|
| **Southern Kazakhstan (Almaty & Zhetysu regions)** | 56.5% of pastureland underused in the context of limited water access; 55 groundwater samples; ≈40% exceeded the study mineralization benchmark; livestock water demand 53,735 × $10^3$ $m^3$/yr | Hydrogeological assessment and groundwater-resource prioritization; treatment need depends on source chemistry | Direct Kazakhstan evidence for the scale of the water-access problem and groundwater heterogeneity; supports site-specific treatment selection rather than a universal BWRO prescription | [9] |
| **Western Kazakhstan (Mangystau Region)** | Regional freshwater deficit and major investment in desalinated municipal supply | Kenderli seawater reverse-osmosis plant; design capacity 50,000 $m^3$/day; commissioned in 2025 | Demonstrates national-scale RO implementation in an arid western region; scale and source differ from decentralized pastoral BWRO | [91,92] |
| **Western Kazakhstan (groundwater irrigation zones)** | Spatially variable groundwater quality; some zones suitable for irrigation and others more saline | GIS, hydrochemical profiling and Sentinel-2-supported spatial assessment | Supports geographically differentiated treatment planning and source prioritization rather than uniform treatment | [93] |
| **Uzbekistan (Ferghana Valley, Karakalpakstan)** | Potential natural fresh/brackish groundwater 27.6 $km^3$/yr; usable groundwater ~0.5 $km^3$/yr; agriculture uses ~90–91% of water | National water-resource planning and water-saving policy; no specific BWRO+UV pastoral field installation is claimed from this source | Useful for regional resource context and policy comparison; technology-transfer claims require separate project-level evidence | [94] |
| **Kyrgyzstan (rural highlands)** | Rural water-supply and sanitation access; climate-resilient infrastructure needs | World Bank Sustainable Rural Water Supply and Sanitation investments: water-supply subprojects, storage, metering, sanitation and institutional strengthening | Expected reach of 94 villages and ~200,000 people; implementation/governance analogue, not evidence for a specific solar-BWRO+UV train | [95] |
| **Australia (Outback / New South Wales)** | Remote arid community; brackish bore water up to ~3,500 mg/L TDS; off-grid solar context | PV-powered hybrid UF + RO/NF field system at White Cliffs | Produced drinking-quality water; reported specific energy consumption 2–8 kWh/$m^3$ depending on conditions; strong technical analogue for remote solar desalination | [71,72] |
| **Middle East / MENA (Gulf States, Jordan)** | Arid-region desalination with strong dependence on salinity, energy source, process scale and local economics | Conventional and renewable-energy-powered desalination; SODIS as a separate small-volume disinfection option | Provides energy/cost benchmarks for desalination and evidence for solar disinfection, but not a single directly transferable pastoral configuration | [35,52,96] |

| Region / Country | Problem context | Technology applied | Key findings and transferability to arid pastoral water supply | Key sources |
|---|---|---|---|---|
| **Sub-Saharan Africa (Uganda, Ethiopia, Kenya)** | Rural and peri-urban communities with limited safe-water access and high solar availability | SODIS and improved solar-disinfection systems | Field/review evidence supports microbial water-quality improvement and, in some settings, reduced diarrhoeal disease; scale is not comparable to livestock water demand | [97,98] |

*Notes: BWRO = brackish water reverse osmosis; NF = nanofiltration; UF = ultrafiltration; SODIS = solar water disinfection; TDS = total dissolved solids. Reference numbers correspond to the numbered reference list in the main manuscript.*
